# Origins and breadth of pairwise epistasis in an α-helix of β-lactamase TEM-1

**DOI:** 10.1038/s41467-026-70627-5

**Published:** 2026-03-17

**Authors:** André Birgy, Clément Roussel, Harry Kemble, Jimmy Mullaert, Karine Panigoni, Audrey Chapron, Mélanie Magnan, Hervé Jacquier, Simona Cocco, Rémi Monasson, Olivier Tenaillon

**Affiliations:** 1 Infection Antimicrobials Modelling Evolution — IAME, Université Paris Cité, Université Sorbonne Paris Nord, INSERM, Paris, France; 2 Laboratoire de Microbiologie, Hôpital Robert Debré, AP-HP, Paris, France; 3 Laboratoire de Physique de l’Ecole normale supérieure, ENS, Université PSL, CNRS, Sorbonne Université, Université Paris Cité, Paris, France; 4 Cancer et Génome, Université Paris-Saclay, UVSQ, Institut Curie, Saint-Cloud, France; 5 Institut Cochin, Université Paris Cité, CNRS, Inserm, Paris, France; 6 Service de Bactériologie, Hôpitaux Universitaires Henri Mondor, AP-HP, Créteil, France; 7 Present Address: Chimie Biologie et Innovation, ESPCI Paris, Université PSL, CNRS, Paris, France

**Keywords:** Evolvability, Antimicrobial resistance, Evolutionary genetics, Experimental evolution

## Abstract

The effect of mutations in a protein may depend on the presence of others—a phenomenon known as epistasis. Epistasis plays a key role in evolution and complicates predictions of mutational effects, as effects can be context-dependent. Yet, despite its importance, the mechanistic basis of epistasis remains poorly understood. To better characterize epistasis, we focused on an 11-residue α-helix in TEM-1 β-lactamase and constructed a comprehensive library of over 14,000 double mutants. Fitness and minimum inhibitory concentration, two contrasted measure of protein efficiency, reveal consistent widespread epistasis. A non-linear two-state protein stability model in which destabilizing, neutral, or stabilizing mutations contribute additively to the stability phenotype, largely explain the data. Most epistatic effects are consequently predictable from single-mutation effects. However, systematic deviations from the model occur when both mutated residues directly interact in the 3D structure—a fold conserved across distant TEM-1 homologs. We therefore investigated the predictive power of statistical models trained on distant homologous sequences and found that they could partially recover the observed epistatic interactions. Our results, built on a short structural element of a protein, shed light on multiple determinants of the epistatic landscape that have shaped the evolutionary trajectory of β-lactamase proteins over long timescales.

## Abstract (web summary)

Analyzing fitness and function of 14,000 double mutants in an α-helix of TEM-1 β-lactamase shows that widespread epistasis is largely explained by a two-state stability model, consistent with constraints inferred from distant homologous proteins.

## Introduction

Sequences of the first proteins triggered the emergence of molecular evolution and bioinformatics in the 1960s^1^. Yet, more than 60 years later, despite a massive number of available protein sequences and a pressing demand from human genetic disease and synthetic biology, the prediction of nonsynonymous mutation effects remains a challenging task.

Over the last decade, two independent approaches have offered new perspectives on the study of nonsynonymous mutation effects. Experimentally, protein deep mutational scans, in which the impacts of all possible single amino acid changes in a protein are investigated, have gained momentum allowing to study not only single mutants but also multiple mutants^2^. At the bioinformatics level, massive protein databases have allowed the use of Multiple Sequence Alignment (MSA) to infer the amino acids that are tolerated or not at a site^3,4^. Interestingly, experimental and data-driven approaches revealed immediately that mutation impact could vary with genetic background^5–7^. It was for instance shown that as little as a single mutation could change quite drastically the impact of many other mutations throughout a protein^5,8^. These observations called for a more comprehensive understanding of mutations’ effects and especially of their interactions.

**Fig. 1:**
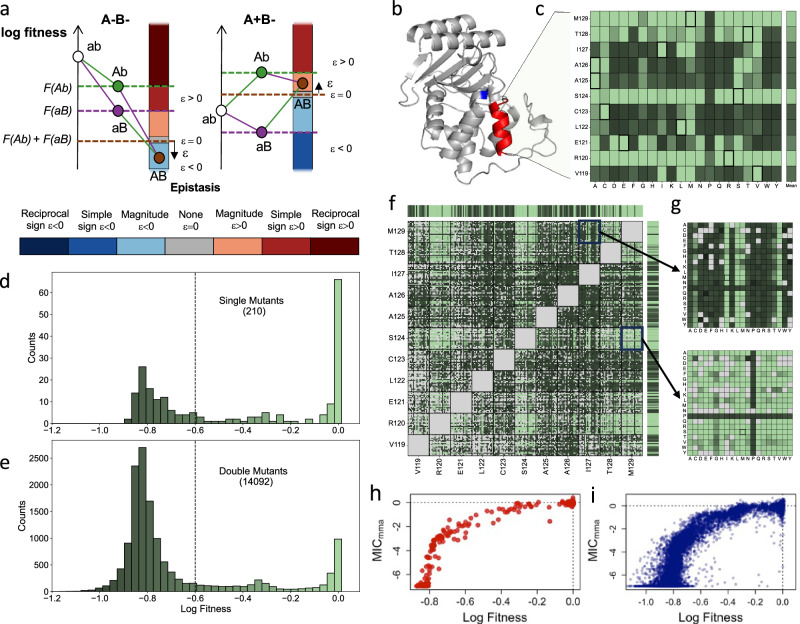
Single and double mutants’ log-fitness effects. **a** Pairwise epistasis measures the deviation of the observed log-fitness of a double mutant from the sum of the log-fitness of its single constituent mutants (F(x) refers in the figure to the log-fitness value of genotype x). It can also be qualitatively categorized as magnitude, sign, and reciprocal sign as well as positive or negative. The figures illustrate how this categorization functions in the case of a pair of deleterious mutations on the left and a pair including a deleterious (b to B) and a beneficial mutation (a to A) on the right (**b**) 3D structure of β-lactamase TEM-1. In red the α-helix of interest, and in blue the Serine residue of the active site. **c** The effects on the log-fitness of all single mutants per residue. Color scale is given in (**d**, **e**), squared amino acid correspond to the wild type sequence. **d**, **e** Distribution of log-fitness effects. Below the dotted line, mutants are considered non-functional. **d** For single mutants. **e** For double mutants. **f** Log-fitness of the double mutants with missing data in light gray. Color scale is given in (**d**, **e**). **g** Zoom on the double mutant log-fitness involving residues I127 and M129 on top and S124 and M129 at the bottom. MIC value, measured as a log2 change in concentration compare to wild type against log-Fitness for single (**h**) and double mutants (**i**). Source data are provided as a Source data file.

Epistasis refers to the context-dependency of mutation effects. In population genetics, pairwise epistasis refers more precisely to mutation interactions that translate in non-additivity of log-fitness effects. In that context, the fitness of a mutant relative to wild type captures the relative change in frequency of that mutant compared to wild type in one generation. Consequently, positive log-fitness reflect the mutation is beneficial, negative that it is deleterious, and null that it is neutral. Epistasis between mutation A and B can then be quantitatively estimated as the deviation between the observed log-fitness variation of the double mutants with respect to WT, AB, and the sum of the log-fitness variations of both individual mutations (A and B) (Fig. 1a). Under this strict definition, epistasis has been predicted to impact significantly many facets of evolution^9^, from the evolution of mutation rate and recombination^10^, to the diversity of adaptive paths and the repeatability of adaptation^11^. These undoubtful significant consequences of epistasis now call for an integrated and mechanistic understanding of epistasis causes.

An integrated vision of epistasis may be developed from a top-down perspective, with phenomenological models that capture its global properties. These models can infer epistasis behaviors from purely statistical properties of sets of interacting mutations^12,13^, or more mechanistically assuming the existence of a genotype to phenotype to fitness map^14^. Interestingly the later have shown that all forms of epistasis mentioned in Fig. 1a can emerge from a simple nonlinear mapping of phenotype to fitness even if the effect of mutations on phenotypes are additive. For instance, all possible forms of pairwise epistasis are observed in the Fisher Geometric Model^14–17^, a smooth singled peaked phenotypic landscape in which fitness is a Gaussian function of the distance to the optimal phenotype. These observations motivated the research of a simple phenotype affected by mutation in an additive manner whose non-linear connection to fitness could explain globally the pattern of epistasis observed. Accordingly, analysis of large datasets of protein’s mutants uncovered the statistical existence of such underlying additive phenotype^18,19^.

As proteins generally operate in a folded state, mutations’ impacts on protein have mainly been investigated through their effects on that fold or its affinity with a substrate. For epistatic interactions, two mutually non-exclusive mechanistic visions have emerged. With compensatory mutations, characterized by two mutations with deleterious effects when considered individually, that, when combined, outcompete at least a single mutant, the idea of key-lock local interactions emerged. Alternatively, the existence of mutations with a global impact on protein stability^8^ hinted that the cooperative nature of protein stability could also result in epistatic effects, this time at a more global level^20–22^. The extent of both types of interactions and the overall prevalence of epistatic interactions remains, however, unclear.

The protein folds that likely underpin these epistatic interactions have also been shown to generate coevolutionary signals along the protein sequence over long evolutionary timescales. These signals are so robust that they can be leveraged to predict protein structures solely from the analysis of MSA of distant homologs, forming a cornerstone of the highly successful AlphaFold^23^ structure prediction software. Furthermore, these coevolutionary couplings can be employed to predict the effects of mutations within genes, predictions that have been validated through experimental deep mutational scans and studies of polymorphisms. However, a critical question remains: how well do these coevolutionary patterns correlate with precise experimental measurements of epistasis? Addressing this will require direct comparisons between coevolutionary predictions and experimental epistasis data.

Numerous deep mutational scan surveys have delved into the realm of epistasis (for instance^24–27^. It is crucial, however, to highlight the limited reliance on cellular fitness estimates within these studies. In most instances, the assay’s readout serves as a surrogate for gene function, be it fluorescence for GFP, direct or indirect measures of protein binding as well as biochemical reporters of gene activities. Take β-lactamases, for example; these enzymes catalyze the degradation of β-lactam antibiotics like amoxicillin, and the impact of mutations is frequently assessed through their effects on the minimum inhibitory concentration (MIC), i.e., the minimum concentration preventing growth^28^. While all these metrics hold relevance, their units possess a degree of arbitrariness. Recognizing that changes in measurement units can influence the quantification of epistatic interactions, we have opted here to compare these measures of gene function effect with a fitness-based approach.

Beyond its relevance for quantifying epistasis, the study of both MIC and fitness provides complementary insights, as each metric captures distinct aspects of the protein’s constraints. Fitness reflects the evolutionary trajectories of mutants, it reflects the mutants relative change in frequency in one generation, while MIC, which reflects the concentration at which growth is substantially inhibited, highlights functional properties of the protein. The distinction is particularly evident in mutants with MIC values below the concentration used to measure fitness. For these mutants, fitness is universally minimal, as they are evolutionarily destined to disappear immediately. However, their MIC values vary substantially, offering a window into their functional impacts. This divergence raises an intriguing question: could epistasis measured through a functional metric like MIC provide additional insights into the long-term evolutionary dynamics of proteins compared to epistasis derived from an evolutionary metric like fitness?

To investigate the molecular determinant of epistatic interactions, we generated a comprehensive library of more than 14,000 single and double mutants within an α-helix of β-lactamase TEM-1 evolving under the selective pressure of the commonly used beta-lactam amoxicillin. TEM-1 is a highly successful antibiotic resistance gene present in about 24% of *Escherichia coli* natural isolates^29^. We focused on an 11 amino-acid α-helix, from residue 119 to 129 (Fig. 1b), as α-helices are the most characterized and frequent secondary structure in protein folds. For the sake of generality, we chose an α-helix that is not involved in the active site; it is just a structural component of the enzyme. The more than 14,000 mutants (64 % of all possible double mutants) were analyzed for their impact on protein activity, measured through MIC, as well as through their effects on fitness, allowing a proper estimation of epistasis (Method). We then investigated how a simple biophysical two-state model linking the sequence-dependent phenotype to the fitness accounted or not for the observed epistasis. Finally, to validate the relevance of our measurement of epistasis and its mechanistic interpretation, we used the protein sequence of numerous distant homologs of TEM-1 to predict mutation effect and epistasis through Direct Couplings Analysis (DCA)^30^.

## Results

### Fitness and MIC of mutants in an alpha helix

**Fig. 2:**
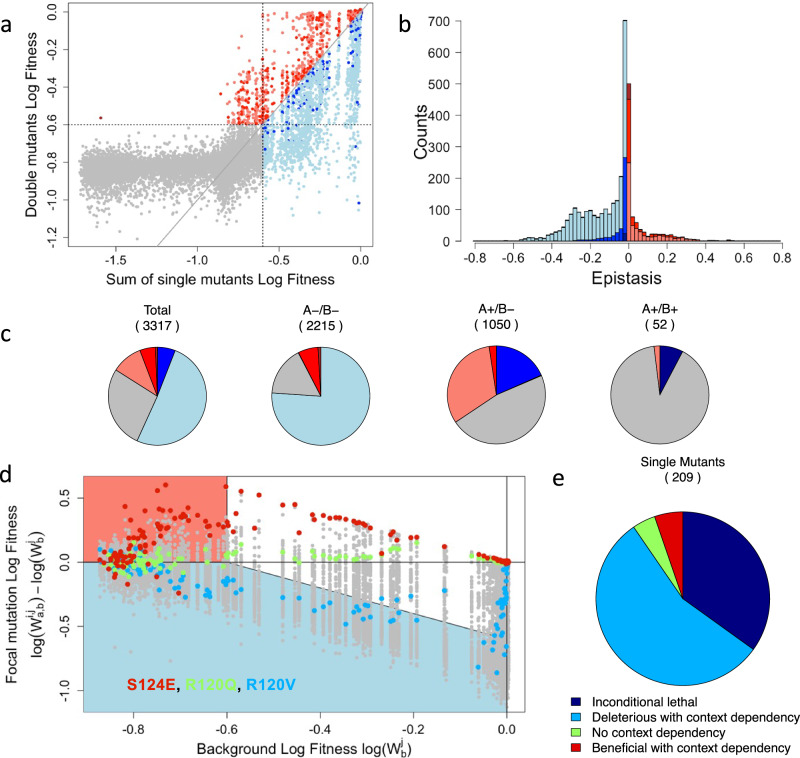
Pairwise epistasis. **a** Log-fitness of effects of double mutants, against the sum of the single mutants’ log-fitness. Gray mutants of observed log-fitness and predicted log-fitness based on single mutants lower than −0.6 cannot give reliable values for the epistasis. The colors of the other points represent the form of epistasis detected using the color code defined in Fig. 1 (**a**). **b** Distribution of epistasis using the same color code, excluding mutants with non-measurable epistasis; histograms are stacked. **c** Categorization of epistasis for all mutations, pairs of deleterious (A−/B−), pairs involving one deleterious and one beneficial (A+/B−), or pairs of beneficial (A+/B+). **d** Relative log-fitness effect of all mutations against the log-fitness of the different backgrounds in which they were found. The values for three focal mutations, L122A, R120K, and S124E, are highlighted in blue, green, and red, respectively. Blue shaded area corresponds to double mutants with fitness effects below the threshold, salmon shaded area corresponds to double mutant with log-fitness value higher than the threshold despite having a single mutant below it. **e** The fraction of mutations falling into unconditionally inactivating, deleterious with context-dependency, no context dependency, and beneficial with context-dependency is presented. Source data are provided as a Source Data file.

To infer fitness of the library of mutants, a competition experiment was performed using a concentration of 8 g/l of amoxicillin. This concentration is a fourth of the MIC of the wildtype genotype. Cells, grown in exponential phase without antibiotics, were diluted in MH broth with amoxicillin and diluted 32-fold into fresh media when reaching an optical density of 0.2. This process ensured a stable exponential growth and antibiotic selective pressure. As a mutant’s change in frequency relative to the wild type sequence can be directly converted to a fitness value (see methods and Supplementary Note 1), we could compute the distribution of log-fitness for all single and double mutants of the dataset from that bulk experiment. The distribution of log-fitness effects of single mutants had two major modes, including one with close to 47% of mutants corresponding to an inactivation of the gene function (log-fitness < −0.6), mutation we qualify later as lethals (Fig. 1c, d and Supplementary Table 1). As the log of 0.5 is −0.69, a log-fitness of less than −0.6 corresponds to mutants that have failed to divide in the presence of the antibiotic (fitness 0.5 relative to a dividing strain). This high prevalence of lethal mutations suggested an overall important role of that α-helix. The different residues had nevertheless very different patterns, with four sites permissive to mutations, while the others were much more sensitive (Fig. 1c). As expected, proline, which is known to be incompatible with α-helix structure, was lethal or close to (log-fitness < −0.54) for the enzyme function at all sites (Fig. 1c). The distribution of double mutant effects appeared to be as well characterized with two major modes, with an even more significant fraction of loss of function genotypes (78%) (Fig. 1e, f). A dominance effect emerged (Fig. 1f, g): mutant combinations including a lethal mutation were lethal. Out of the 10,000 double mutants involving at least a lethal mutant (10,006 for replicate 1, 10,087 for replicate 2), about 1% had a log-fitness higher than −0.5 (95 for replicate 1, 105 for replicate 2) and all combinations of lethal mutations resulted in a lethal double mutant except one that was close to being lethal in one replicate (log-fitness < −0.56 and lethal in the other replicate, Fig. 2a). This general dominance effect suggests that the key-lock epistatic compensations, characterized by two deleterious mutations, which, when combined, outcompete at least a single mutant, are rare in the α-helix under study. It also clarifies the partial success of methods based on residue conservation^3,4^ to predict mutation effect: significant effects, such as inserting a proline within an α-helix are effectively context-independent.

We also quantified the minimum inhibitory concentration (MIC) of the mutants using three distinct metrics (Methods, Supplementary Note 2, Supplementary Fig. S1). First, we determined the log2-transformed antibiotic concentration that caused 75% mortality among the mutants, MIC_75_ (see “Methods”). Second, we applied a moment-matching approach to estimate the log2 concentration at which mutant counts shifted from high to low, MIC_mma_ (see “Methods”). Lastly, we integrated these two metrics via principal component analysis MIC_combined_ (see “Methods”). For each metric, deviations from the wild-type values were calculated, for these metrics as well as for fitness the correlation between replicates was high (Supplementary Fig. S2).

The MIC distributions for single and double mutants qualitatively mirrored those observed for fitness. Similarly, the qualitative patterns in the distributions of effects for single and double mutants aligned with expectations, consistent with the strong correlation between MIC and fitness estimates (Pearson’s *r* > 0.88, Spearman’s *ρ* > 0.94 for single mutants (*n* = 209) (later on r will stand for Pearson’s *r* and *ρ* for Spearman’s); *r* > 0.87, *ρ* > 0.78 for double mutants (*n* = 14092), Figs. 1h, i, Supplementary Table 2 and Supplementary Fig. S3). However, many mutants classified as lethal in the fitness assay—due to their inability to replicate in the presence of 8 mg/L of antibiotic—exhibited non-minimal MICs. As a result, a substantial proportion of single and double mutants displayed non-minimal MICs, comprising 86% and 74%, respectively (Supplementary Table 1).

### Wide spread epistasis in an alpha helix

We next focused on quantifying epistasis (Fig. 2, Methods, Supplementary Note 3), prompted by the observation that the log-transformed fitness and MIC of double mutants often deviated substantially from the values expected under additivity—defined as the sum of the log-transformed fitness (Fig. 2a) or MIC of the corresponding single mutants (Supplementary Fig. S4). Epistasis could be measured with high resolution only in cases where both the single mutants and the double mutant were non-lethal. Restricting our dataset to this subset, epistasis was highly correlated between replicates (Spearman’s *ρ* > 0.88, *n* = 2897) but correlated to a lesser extent between fitness and MICs epistasis measures (Spearman’s ρ 0.62 > ρ > 0.54, (*n* = 7950, 8047, 8027) Supplementary Tables 3, 4, 5 and Supplementary Fig. S2e S2f). Overall, we observed that the distribution of epistasis was broad and centered near zero but exhibited a bias toward negative values (Fig. 2b and Supplementary Fig. S5), consistent with findings from studies using protein function proxies e.g., binding affinities or fluorescence^24,27^. Nevertheless, instances of pronounced positive epistasis were detected, particularly in cases where a deleterious mutation was paired with a beneficial one (Fig. 2c).

To investigate the determinant of epistasis, we looked at how the effect of a focal mutation was affected by the effect of the other mutations it was associated to. For a given focal mutation, A, we plotted in Fig. 2d the effect of the double mutants AB minus the effect of the single mutant B (called focal mutation relative effect) versus the effect of single mutants B (called background effect). This illustrates how the impact of mutation A is dependent on the fitness of the background in which it appears. In this figure, for log-fitness, the white area corresponds to mutants with high resolution on log-fitness for double mutants AB and single mutant B (log-fitness > −0.6). The blue region corresponds to lethal double mutants. And finally, the orange area corresponds to lethal single mutant B but where the double mutants AB have log-fitness greater than -0.6. Due to the high resolution of log-fitness in the white area, we are mainly interested in the patterns that mutations exhibit in this area. These plots and their equivalent for the various MIC measurements (Supplementary Fig. S6) reveal very contrasted and structured patterns for the different mutations that we grouped in four distinct categories (Fig. 2e).

For fitness, among the 209 possible single mutants, 72 (34%, Supplementary Table 6) are lethal across all backgrounds (the single mutants and all the double mutants, including these single mutants, having a log-fitness lower than -0.6). Due to the resolution limits of our assays, we could not draw significant conclusions about these loss-of-function mutations. Of the remaining mutations, 8 (4%) showed no significant interaction between the effect of the focal mutation and the fitness of the genetic background, corresponding to a flat line in Fig. 2d (e.g., mutation R120Q, green points). These mutations had minimal impacts on log-fitness (<0.25%). All other mutations exhibited effects that varied depending on the fitness of the genetic background in which they were introduced. In 117 cases (56%), the effect of the mutation became more deleterious as the background fitness decreased, a hallmark of negative epistasis (e.g., mutation R120V, blue points). Finally, 12 mutations (6%) that were marginally beneficial in the ancestral background exhibited positive epistasis, with their effects becoming more advantageous in combination with deleterious mutations (e.g., mutation S124E, red points). For MIC, the patterns were highly consistent. As expected, some fully lethal mutants at the 8 mg/l concentration had a none minimal MIC, consequently, the number of full lethal mutants consequently decreased from 72 to 20–23, while the number of deleterious mutants with signature of negative epistasis increased from 117 to 157–160 (Supplementary Table S3). The number of mutations with positive epistasis remained stable. Mutations without a clear epistasis pattern increased to ~20, likely due to lower resolution in MIC measurements.

Remarkably, excluding universally lethal mutations, approximately 90% of mutations exhibited clear context dependencies shaped by background fitness. This consistency suggests the involvement of a global force, such as protein stability, in structuring these dependencies.

### A two-state model is predictive of epistasis

**Fig. 3:**
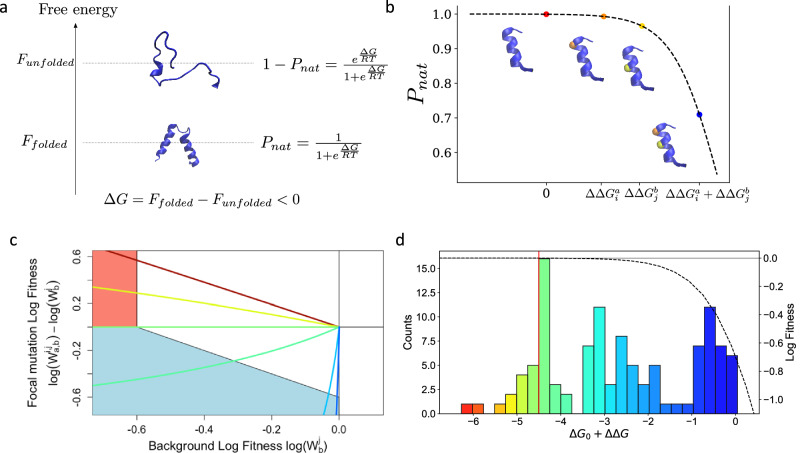
Stability and context-dependency. **a** Stability model. ${P}_{{nat}}$ is the probability that the protein folds. **b** Effects of the mutations on the stability. Black dotted line corresponds to ${P}_{{nat}}$. Red dot corresponds to the wild-type. Orange dot corresponds to a single mutation on the α-helix, with $\Delta \Delta {G}_{i}^{a}$. Yellow dot corresponds to a single mutation on the α-helix, with $\Delta \Delta {G}_{j}^{b}$Blue dot corresponds to double mutations on the α-helix, with $\Delta \Delta {G}_{i}^{a}+\Delta \Delta {G}_{j}^{b}$. Mutations are considered as additive in $\Delta \Delta G$. However, this results in non-additive effect in ${P}_{{nat}}$. **c** The relationship between background log-fitness and mutant’s relative log-fitness predicted by the model of stability is presented. The protein modeled has a free energy of −4.55 kcal mol^−1^, and the impact of mutations, $\Delta \Delta G$, is −2, −0.5, 0, 0.5, 2 and 3 kcal mol^−1^ from red to blue. **d** Histogram of the $\Delta \Delta G$ estimated. Red line corresponds to $\Delta {G}_{0}$. Black dashed line corresponds to ${P}_{{nat}}$ as a function of $\Delta {G}_{0}+\Delta \Delta G$. Source data are provided as a Source data file.

A key paradigm in protein analysis posits that most residues primarily contribute to maintaining the functional fold of the protein, with mutations at these sites predominantly affecting stability rather than activity^31^. Protein stability is often described using a two-state model, representing a functional folded state and various nonfunctional unfolded states^20,32^ (Fig. 3a, b). The probability *P*_*nat*_ that a protein adopts its correct functional structure depends on the free energy of its amino-acid sequence, which can be defined as the sum of the wild-type sequence free energy, $\Delta {G}_{0}$, and of the modifications of that free energy due to the mutations, ∆∆*G*:

1 $${P}_{{nat}}\left({mut}\right)=\frac{1}{1+{e}^{\frac{\Delta {G}_{0}+\Delta \Delta G}{{RT}}}}$$

One of the main hypotheses in this model is the additivity of ∆∆*G* upon multiple mutations affecting different residues, for instance:

2 $$\Delta \Delta {G}_{i,j}^{a,b}=\Delta \Delta {G}_{i}^{a}+\Delta \Delta {G}_{j}^{b}$$

where $\Delta \Delta {G}_{i,j}^{a,b}$ represents the energy change resulting from the double mutations introducing amino acid *a* and *b* at sites *i* and *j* respectively, $\Delta \Delta {G}_{i}^{a}$ denotes the energy change caused by a mutation leading to amino acid *a* at site *i* and $\Delta \Delta {G}_{j}^{b}$ corresponds to the one associated with a mutation leading to amino acid *b* at site *j*. Of note, additivity of $\Delta \Delta G$ does not exclude epistasis due to the non-linearity of ${P}_{{nat}}$ relative to $\Delta \Delta G$.

To explore whether protein stability could explain the observed patterns of epistasis, we incorporated the two-state model (Eq. 1) as a fitness framework^18,20^, under the assumption that the fraction of folded protein, ${P}_{{nat}}$, is directly proportional to fitness, *W* (Methods). Since our focus is on relative log-fitness with respect to the WT, the stability-based log-fitness of a mutant can be calculated as:

3 $${{\mathrm{ln}}}\left(\frac{W}{{W}_{{WT}}}\right)={{\mathrm{ln}}}\left(1+{e}^{\frac{\Delta {G}_{0}}{{RT}}}\right)-{\mathrm{ln}}\left(1+{e}^{\frac{\Delta {G}_{0}+\Delta \Delta G}{{RT}}}\right)$$

Remarkably, depending on the mutant $\Delta \Delta G$, this model produces patterns of log-fitness effects according to background log-fitness similar to the one observed in the data (Fig. 3c).

We resorted, therefore, to estimate quantitatively the model’s parameters, $\Delta \Delta G$ and $\Delta {G}_{0}$, from the log-fitness of single and double mutants (Method, Supplementary Note 4). Since log-fitness measurements are reliable only above a threshold of −0.6, we retained 111 single mutants (53% of the total) with log-fitness values exceeding this threshold. For each pair of selected single mutants, the corresponding double mutant was included if its log-fitness was experimentally measured, though values were thresholded at −0.6. The stability model was similarly constrained at -0.6 during parameter inference. Including thresholded lethal double mutants improved the estimation of $\Delta \Delta G$. Using this approach, we inferred $\Delta {G}_{0}$ = −4.5 kcal mol^−1^ (Fig. 3d). To ensure reproducibility, the experiments were conducted using two biological semi-replicates (the same library evolved in parallel). For both replicates, the inferred $\Delta \Delta G$ values were highly correlated (*r* > 0.99, Supplementary Table 7 and Supplementary Fig. S2g and S2h).

**Fig. 4:**
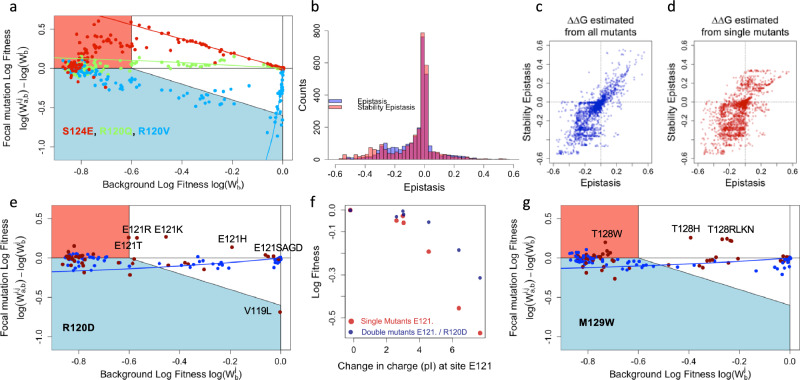
Stability and epistasis. **a** The stability model reproduces the observed context dependency of mutation effets. The lines represent the fit of the model for the three mutants from Fig. 2d. Due to the resolution of our experiments, the lines are valid only in the white area. **b** Observed and inferred distribution of epistasis. In blue is the distribution of epistasis as presented in Fig. 3b, and overlaid on it in red is the distribution of epistasis obtained with the fitted stability model. The correlation between observed epistasis and the one derived the stability model with ∆∆G estimated using all mutants (**c**) or just the single mutants (**d**). **e**–**g** Deviations from the stability model. Relative log-fitness according to background fitness for mutants R120D (**d**), and M129W (**g**). Red dots represent distant residues in the protein 3D structure and blue dots nearby residues. **f** Amino acid changes at residue 121 are counter selected according to their impact on charge (red dots), an effect that is alleviated by the addition of mutation R120D (blue dotes) that impact charge in the other direction. Source data are provided as a Source data file.

The stability model reproduces well the experimental log-fitness of all the selected single (*ρ* > 0.98, *r* > 0.999 (*n* = 110)) and double mutants (*ρ* > 0.92, *r* > 0.93, (*n* > 3998)) Supplementary Table 8). The above correlation is an improvement with respect to the one (*ρ* < 0.89, *r* < 0.88, Supplementary Table 9) obtained when neglecting epistasis, i.e., assuming that the log-fitness of double mutations is the sum of the ones of simple mutations. Moreover, as shown in Fig. 4a the stability model captures the overall dependency of the fitness of a double mutant from the fitness of the background simple mutant. Finally, Fig. 4b shows that, for fitness, it reproduces the shape and breadth of the distribution of epistasis, with correlation *ρ* = 0.81, *r* = 0.76 (*n* > 3300) between observed and predicted epistasis (Fig. 4c and Supplementary Table 8). Our results are also consistent with previous experiments: R120G is known to have a stabilizing effect^33,34^, and this effect is indeed captured by the model, with $\Delta \Delta G$ = -1.45 kcal mol^−1^ (negative $\Delta \Delta G$ corresponds to stabilizing mutation).

Building on the ability of the two-state model to accurately capture both the background dependency of mutants and epistasis, we next assessed its performance in predicting double mutation effects and epistasis when $\Delta {G}_{0}$ and $\Delta \Delta G$ parameters were inferred using data from single mutations only. This approach effectively reduces the number of data points used to train the model from more than 4000 to 111 (for fitness). As shown in Fig. 4d, the model achieves a notable Spearman’s correlation of 0.6–0.7 in predicting epistatic effects. The variability in Pearson’s correlation values arises from the imprecise estimation of $\Delta {G}_{0}$, driven by flat directions in the log-likelihood of the two-state model when fitted solely to single mutation data. Notably, as illustrated by the comparison of Fig. 4c, d, predictions of positive epistasis are less accurate when model parameters are derived exclusively from single mutations. This limitation is attributable to the stabilizing effects ($\Delta \Delta G$ < 0) of certain single mutants, which yield log-fitness values close to 0. When only single mutants are considered, these stabilizing mutations are inferred as $\Delta \Delta G$ = 0, as their effects become apparent only when double mutants are included in the analysis.

A similar approach was employed to evaluate how well the stability model aligns with different MIC metrics. MIC values span a broader range compared to log-fitness estimates, as many mutants with minimal fitness at the tested concentration still exhibit non-minimal MICs. While 111 mutations displayed non-minimal log-fitness, 179–182 mutants had non-minimal MIC values. The best-fit $\Delta {G}_{0}$ values ranged from −4.0 to −2.35 kcal mol^−1^, depending on the MIC metric used. Correlations between inferred $\Delta \Delta G$ values across replicates were very high, exceeding 0.99 for both Spearman’s and Pearson’s statistics (Supplementary Table 7).

The stability model demonstrated strong predictive accuracy for MIC effects. For single mutants, the correlation between predicted and observed MIC values was very high (*r* > 0.99, *ρ* > 0.89, *n* > 178), and for double mutants, it remained robust (*r* > 0.93, *ρ* > 0.92 (*n* > 10,000), Supplementary Table 8). Furthermore, the model effectively captured a significant portion of epistatic interactions (*r* > 0.66, *ρ* > 0.74, *n* > 8000), though the fits were slightly less accurate than those obtained for fitness data despite the larger range of MIC. The disparity was more pronounced when estimating $\Delta \Delta G$ values from single-mutant MICs across a range of $\Delta {G}_{0}$ values. For fitness, Spearman’s correlations between predicted and observed epistasis were relatively high (>0.6), but for MIC, the maximum correlation ranged from 0.16 to 0.47. This reduction in correlation likely stems from the greater noise inherent in MIC measurements, which amplifies the variability in epistasis estimates.

### Deviations from the two-state model are more frequent between physically close residues

Although the two-state model generally reproduces our data well, we next examined the specific site pairs where its predictions deviated most significantly from the observed values. First, when keeping only the residues at less than 6 Å the correlation between experimental and predicted log-fitness with the two-state model decreased to *ρ* = 0.89, *r* = 0.90 (*n* = 1845), while the correlation improved to *ρ* = 0.94, *r* = 0.95 (*n* = 2241) when only distant pairs (>6 Å) were considered. The results were confirmed with predicted epistasis correlating better for distant residues (*ρ* = 0.86 for *d* > 6 Å (*n* = 1886) and *ρ* = 0.76 for *d* < 6 Å (*n* = 1428) for fitness, similar results were obtained for epistasis and MIC estimates, Supplementary Tables 10 and 11). Accordingly, a maximum likelihood model (Supplementary Note 5 and Supplementary Table 12) estimated that the deviations to the two-state model were 1.32 times greater for close pairs of sites (<6 Å) compared to distant ones (>6 Å) for log-fitness (and 1.11 times greater for MIC measurement the difference being presumably due to higher error rate in MIC measurements). This implies that our model explained better interactions between distant sites than between nearby sites, suggesting that for local interactions, alternative forces could be at play.

To further characterize deviations from two-state model predictions, we computed, for each pair of residues *i* and *j*, the mean square error between the experimental log-fitness log(${w}_{i,j}^{a,b}$) and the log-fitness predicted with the stability model log(${\hat{{{\rm{w}}}}}_{i,j}^{a,b}$) (Eq. (3),

4 $${D}_{{ij}}=\sqrt{\frac{1}{{N}_{{ij}}}{\sum }_{a,b}{\left({{\mathrm{ln}}}({w}_{i,j}^{a,b})-{{\mathrm{ln}}}({\hat{{{\rm{w}}}}}_{i,j}^{a,b})\right)}^{2}}$$

where ${N}_{{ij}}$ is the total number of double mutants for which we can calculate the log-fitness according to the stability model for the pair *i*, *j* (Supplementary Fig. S7 for the distribution of ${D}_{{ij}}$). Large deviations ${D}_{{ij}}$ imply that the assumption of linearity of the $\Delta \Delta G$ embedded in the two-state model (Eq. 2) is no longer valid for the corresponding pair. We refer to such pairwise interactions, not captured by the stability model, as idiosyncratic epistasis. For instance, mutation R120D (Fig. 4e, f). and M129W (Fig. 4g) showed signs of both positive and negative epistasis, the positive epistasis being restricted to residues in direct contact. R120D, a marginally costly mutation when associated to distant residues, becomes beneficial when paired to some mutations at the neighboring residue E121. R120D seems to compensate partially the costs associated with changes in charge caused by mutation at the E121 residue (Fig. 4f). The five pairs of sites with the largest idiosyncratic epistasis are: 128–129, 124–128, 123–127, 127–128, and 120–123. Among these five pairs, four correspond to the residues at less than 6 Å (the last one corresponding to a distance of 6.17 Å), comforting that local interactions involve the largest deviation from the two-state model.

### Sequence of TEM-1 homologs can be used to predict mutation effects in TEM-1

At this stage, the analysis of our experimental data suggests that epistasis results largely from a non-linear relationship between the sequence of a protein and its macroscopic fitness, well captured by a two-state model. Moreover, for both fitness and MIC measurements, the deviations to the model are not random and occur preferentially for residues in contact, revealing this time some idiosyncratic epistasis. We next wanted to validate that these observations on epistasis, made by measuring fitness in the laboratory at a given antibiotic concentration or through MIC measurements, could be representative of generic properties of epistasis in the TEM-1 protein family, class A β-lactamases that evolved for millions of years.

To this aim, we trained a model on an MSA built on high-quality homologs of class A β-lactamases cleaned by hand^35,36^ and enriched on SwissProt and TrEMBL^37^ (Method). The main idea is to learn a probability distribution over all the sequences ***a*** of length L from the MSA: sequences with high probability should correspond to putative β-lactamase. Each sequence ***a*** is supposed to be drawn from a Boltzmann distribution $P\left(a\right)=\frac{{e}^{-E(a)}}{Z}$. Once trained to reproduce the amino-acid frequencies and pairwise correlations in the MSA, the distribution P allows us to score all the single and double mutants according to their statistical energies *E(****a****)*. For such model, known as Potts models, *E(****a****)* reads

5 $$E\left(a\right)=-{\sum }_{i=1}^{L}{h}_{i}\left({a}_{i}\right)-{\sum }_{1\le i < j\le L}{J}_{{ij}}({a}_{i},{a}_{j})$$

with fields ${h}_{i}\left({a}_{i}\right)$ and pairwise interactions ${J}_{{ij}}\left({a}_{i},{a}_{j}\right)$. Potts model, used in Direct-Couplings Analysis (DCA)^30,38^ can disentangle direct coevolutionary couplings from indirect ones. The Potts interactions are known to be good predictors of tertiary contacts. In addition, this family of models was successfully used to design functional proteins with limited homology to existing sequences^39^, and for TEM-1, they have been used for predicting fitness effects of single mutations^40^.

To compare the predictions *E(****a****)* and the results of the experiments, we need a proxy to link the two quantities. The most common proxy is the difference of log-scores between the mutant ${{{\boldsymbol{a}}}}_{{{\boldsymbol{mut}}}}$ and the wild-type ${{{\boldsymbol{a}}}}_{{{\boldsymbol{WT}}}}$

6 $${Potts}\left({a}_{{mut}}\right)={\mathrm{ln}}\left(P\left({a}_{{mut}}\right)\right)-{\mathrm{ln}}\left(P\left({a}_{{WT}}\right)\right)=-E\left({a}_{{mut}}\right)+E\left({a}_{{WT}}\right)$$

With that proxy, Potts energies were found to be correlated to MIC^40^, specificity constant ${k}_{{cat}}$^41^, log-fitness^42^, or binding energies^43^. Accordingly, for log-fitness, we found a Spearman correlation *ρ* = 0.84 for the 143 single mutants whose mutations have been observed in the MSA (to avoid relying on some arbitrary thresholding) and *ρ* = 0.73 for the 6632 associated double mutants with measures for all metrics. This has to be compared with the slightly worse results, *ρ* = 0.73 for single mutants and *ρ* = 0.62 for doubles, obtained with the independent model (Supplementary Fig. S8) that only considers conservation of amino acids and not coevolution between sites (same energy as Potts model but without interactions ${J}_{{ij}}({a}_{i},{a}_{j})$). The Potts model, allows to better estimate the effects of the mutations, thanks to the couplings ${J}_{{ij}}({a}_{i},{a}_{j})$ which takes into account the background of TEM-1, instead of having an average global effect consistent across all the class A β-lactamases, as in the case of the independent model. For MIC, we found similar correlations, with 0.80> *ρ* > 0.75 for single mutants and *ρ* ~0.77 for double mutants (Supplementary Table 13).

**Fig. 5:**
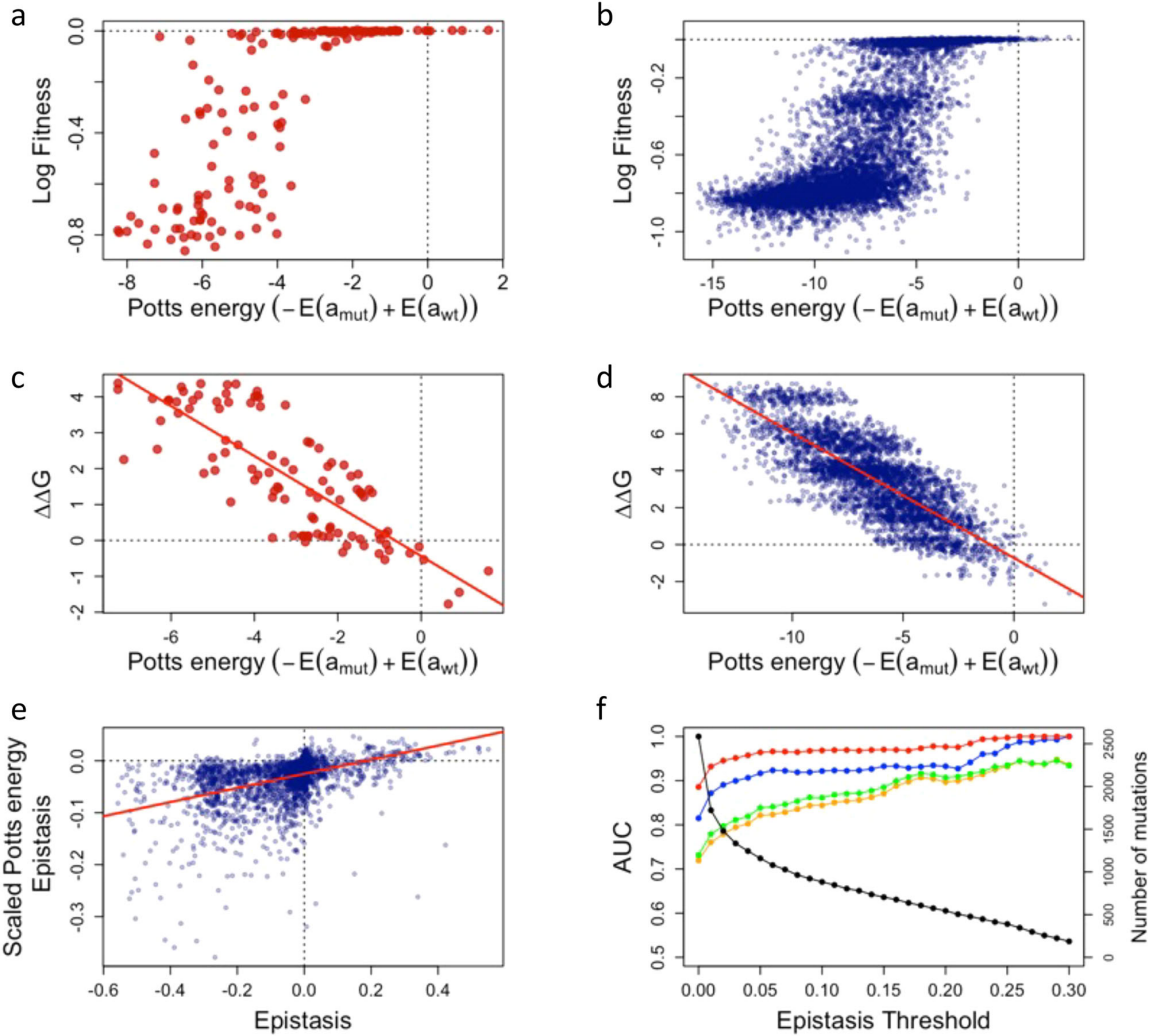
MSA based fitness and epistasis predictions. **a**, **b** Energy from Potts model inferred from sequence data, versus experimental observations for single mutants (**a**, **c**) or double mutants (**b**, **d**). Only mutations present in the MSA are kept (with a different background than TEM-1) and for panel c and d only mutants with estimates of ∆∆G are included. **a** Experimental log Fitness against $-E({a}_{{mu}{t}_{i}^{a}})+E({a}_{{WT}})$ for single mutants. **b** Experimental log Fitness against $-E({a}_{{mu}{t}_{{ij}}^{{ab}}})+E({a}_{{WT}})$ for double mutants. **c** $\Delta \Delta {G}_{i}^{a}$ against$-E({a}_{{mu}{t}_{i}^{a}})+E({a}_{{WT}})$ **d** $\Delta \Delta {G}_{i}^{a}+\Delta \Delta {G}_{j}^{b}$ against $-E({a}_{{mu}{t}_{{ij}}^{{ab}}})+E({a}_{{WT}}).$ **e** Estimated epistasis with Potts energies in the stability model against experimental epistasis. Our predictions capture mostly the sign of the experimental epistasis. **f** AUC against epistasis’ threshold for the different models. We used a threshold for the epistasis, keeping only experimental epistasis above this threshold in absolute value. In red, the predictive power of the sign of epistasis using the stability model relying on all mutants to estimate the $\Delta \Delta {G}_{i}^{a}$ is represented. Blue corresponds to the stability model this time using $\Delta {G}_{0}\,$= −4.5 kcal mol^−1^, and only single mutant fitness. Green corresponds to the predictions derived from the Potts energy rescaled using all mutant fitness measures, and in orange corresponds to a rescaling of Potts energy using only the fitness of single mutants. Source data are provided as a Source Data file.

As shown in Fig. 5a, b the relation between the log-fitness and our Potts energy is highly nonlinear with a characteristic S-shape displaying saturation of the log-fitness both at large and small Potts energy values. As is shown in Supplementary Fig. S9, the S-shape depends, as expected, on the experimental proxy to measure the fitness and changes when using the MIC instead of the relative enrichment at a fixed concentration, accordingly to the non-linear relation between MIC and relative enrichment (Supplementary Fig. S3). For the independent model, the relationship is even more bimodal (Supplementary Fig. S8). Due to the above nonlinear relation between the Potts model energy and the experimentally measured epistasis, the Potts model failed to predict epistasis (*r* < 0.037 (*n* > 2887), Supplementary Table 14) when using directly the energy differences as a measure of epistasis.

7 $${{Epistasis}}^{{Potts}}=	 -E\left({a}_{{mu}{t}_{{ij}}^{{ab}}}\right)-E\left({a}_{{WT}}\right)+E\left({a}_{{mu}{t}_{i}^{a}}\right)+E\left({a}_{{mu}{t}_{j}^{b}}\right) \\=	 {J}_{{ij}}\left({a}_{{mu}{t}_{i}^{a}},{a}_{{mu}{t}_{j}^{b}}\right)$$

Yet, the typical “S” shape between the log-fitness and the Potts model energies is reminiscent of the relationship described by the stability two-state model. By directly comparing the Potts energies to the two-state model $\Delta \Delta G$ parameters, we found high correlations (for fitness *ρ* ~0.80, compared to *ρ* ~0.60 for the independent model, (*n* = 141) Supplementary Table 15), that were associated with much more linear relationship as shown in Fig. 5c, d. This suggests that the Potts model energies can be seen as an equivalent of $\Delta \Delta {{\rm{G}}}$ in the two states model:

8 $$\Delta \Delta {G}_{i}^{{a\; Potts}}=\gamma {Potts}\left({a}_{{mu}{t}_{i}^{a}}\right)=\gamma {h}_{i}\left({a}_{{mu}{t}_{i}^{a}}\right)$$

for single mutants, and for double mutants, we replace $\Delta \Delta {G}_{i}^{a}+\Delta \Delta {G}_{j}^{b}$ by:

9 $$\Delta \Delta {G}_{{ij}}^{{ab\; Potts}}=	 \gamma {Potts}\left({a}_{{mu}{t}_{{ij}}^{{ab}}}\right) \\=	 \gamma \left({J}_{{ij}}\left({a}_{{mu}{t}_{i}^{a}},{a}_{{mu}{t}_{j}^{b}}\right)+{h}_{i}\left({a}_{{mu}{t}_{i}^{a}}\right)+{h}_{j}\left({a}_{{mu}{t}_{j}^{b}}\right)\right)$$

Accordingly, Potts model energies seem to encapsulate a component of the impact of mutations on protein stability. However, because these energies do not directly represent the absolute $\Delta \Delta {{\rm{G}}}$ values of mutations, they fail to reliably estimate epistasis. This limitation arises due to the non-linear relationship between $\Delta \Delta {{\rm{G}}}$ and ${P}_{{nat}}$ (Eq. 1), which requires appropriately scaled values. To investigate this, we first rescaled the Potts model energies using the regression coefficient γ (Supplementary Table 16), derived from the regression between single-mutant $\Delta \Delta {{\rm{G}}}$ values and Potts model energies. The rescaled values were incorporated into Eq. 3 using the previously estimated ${\Delta G}_{0}$ value (e.g., ${\Delta G}_{0}\,$= −4.5 kcal mol^−1^ for log-fitness). Restricting our analysis to mutations present in the MSA and with available $\Delta \Delta {{\rm{G}}}$ estimates, the predicted fitness calculated from the rescaled Potts energies demonstrated an ability to predict epistasis: a Spearman’s ρ of 0.44 was found with observed epistasis values (Pearson’s *r* = 0.35, (*n* > 1800). Predictions were even more accurate for MIC metrics (*ρ* ≈ 0.52, *r* ≈ 0.50, *n* > 4200).

To refine this further, we employed a maximum likelihood approach to optimize the scaling and shifting of Potts energies for improved epistasis prediction (Supplementary Tables 17 and 18). Using log-fitness (or MIC) values for either single mutants or all mutants, we optimized $\Delta {G}_{0}^{{Potts}}$ and *γ* (Eq. 8). This optimization revealed that an appropriate rescaling of Potts energies could enhance Spearman’s correlation to as high as 0.52 (*n* > 2300) for log-fitness and 0.56 (*n* > 3800) for MIC. However, as illustrated in Fig. 5e, the model primarily captures the directionality (sign) of epistasis. This was further quantified using the AUC-ROC curve shown in Fig. 5f. An over-presentation of good predictions of the sign of the epistasis (AUC-ROC > 0.5) is observed. The quality of this prediction improves further as we restrict our analysis to pairs of mutations showing higher magnitude of epistasis.

### Sequence of TEM-1 homologs predicts pairs of sites with idiosyncratic epistasis

As mentioned before, without encompassing the non-linearity of the two-state model, the Potts model fails to capture the epistatic effects. Nevertheless, since Potts models are powerful tools to determine contacts between residues in protein structures, we investigated if these models could be predictive of the identified idiosyncratic epistatic interactions we detected. For Potts model, the canonical proxy to measure the interactions between two specific sites is the Frobenius norm of the coupling matrices

10 $${F}_{i,j}=\sqrt{{\sum }_{a,b}{J}_{{ij}}{\left(a,b\right)}^{2}}$$

with the average product correction^44^. The top couplings of this metric are traditionally used to predict the tertiary contacts^38^.

We found that among the five pairs of sites with the largest Frobenius norm, there are three pairs with significant idiosyncratic epistasis: 124–128, 127–128, 128–129. Under the assumption that there is no link between these two quantities, it leads to a *p*-value equal to 0.0036 (Supplementary Note 6). Therefore, the most interacting pairs of sites predicted by the Potts model within the α-helix correspond to the pairs of sites where local idiosyncratic interactions were detected experimentally. This suggests that these interactions lead in the long term to some specific coevolution patterns between pairs of sites that are captured by Potts model. However, these effects are not captured at the scale of the interactions between two specific sites and two specific amino acids, but at the scale of the pairs of sites over all amino acid combinations.

## Discussion

The deep mutational scan we have performed here to study mutation effects in a local alpha-helix of the beta-lactamase TEM-1 reveals that epistasis is pervasive. We found that once we exclude mutations carrying irrevocable loss of function, about 90% of mutations showed some strong signature of epistasis. Interestingly, though we work on a small fraction of the protein, most epistasis does not result from idiosyncratic interactions between sites, and is captured by a global model of stability. In that model, the phenotypic impact of mutant adds up in double mutants but the non-linear translation of phenotype to fitness results in epistasis^18–20^. The functional form of the non-linear mapping between the fitness and the phenotype may reflect the global impact of the mutations on the protein stability, in particular for the secondary structure component under investigation, and on its functionality. The phenotype to fitness mapping therefore reflects the environmental pressure on the activity of the protein, tuned by the experimental conditions, here determined by the antibiotic concentration^18,45,46^.

Using the two-state model and the single and double mutations scan, we could estimate for each single mutant a phenotypic effect in the form of an energy change, $\Delta \Delta {{\rm{G}}}$. Within this model, we could explain both qualitatively and quantitatively a large fraction of the observed epistasis (ρ = 0.81 for Log-fitness). Moreover, as, according to the two-state model, the mutational effects on the phenotype are additive, we could fit the $\Delta \Delta {{\rm{G}}}$ parameters only from the single mutational data, to predict epistasis with a good accuracy, as estimated with a Spearman correlation ranging from 0.6 to 0.7.

The large contribution of this global epistasis we observed despite our focus on a local structure of the protein is remarkable and further emphasizes the importance of this form of epistasis, whose overall relative contribution should only increase as we consider larger fractions of the protein. Accordingly, the study of mutants’ impact on stability among 500 human protein domains^22^, has revealed that a large fraction of mutations impact protein stability. Here, we show that protein stability is a major driver of fitness and MIC epistasis among pairs of mutations. The importance of these macroscopic form of epistasis at the protein level is reminiscent of the negative epistasis found genome-wide in experimental evolution^12,13,47–50^.

Our precise estimates of log-fitness allowed us to identify some deviations from the two-state model. Interestingly, there was also some consistency in these deviations that were more likely to occur between residues in direct contacts in the protein structure. We found for instance some examples of local interactions linked to charge conservation. Deviation from the additivity at the phenotypic level may generate these deviations from macroscopic epistasis. We would like to point out that our alpha helix is not included in the active site of the protein. We believe that the two-state model would be less predictive for sites included in the active site, where activity would predominate over global epistasis. Accordingly, a study focusing on epistasis within the active site of another beta-lactamase^51^, CTX-M-14, revealed a more complex contribution of stability to epistasis: some destabilizing mutations were found to be generic sources of positive epistasis contrary to predictions of the two state model. However, we estimate that for a majority of sites, the global epistasis derived from the two-states model dominates.

Both global epistasis and deviation from it seem to be connected to the 3D structure of the alpha-helix under investigation, either through the impact of mutations on protein stability or through contacts between the residues. Because such structure is highly conserved, we then questioned whether the determinants of epistasis were conserved enough to be detected from the analysis of MSA of distant homologs that share the same fold. Interestingly, both the signature of the macroscopic model and the patterns of deviations were recovered through the integration of MSA in the Potts model. First, the estimated $\Delta \Delta {{\rm{G}}}$ correlated linearly with the Potts model mutation energy predictions. However, because macroscopic epistasis results from a precise non-linear mapping of phenotype to fitness, the Potts model estimates of $\Delta \Delta {{\rm{G}}}$ had to be rescaled before being inserted in the two-state model to have some predictive power on the observed epistasis (mostly on the sign of epistasis). Second, pairs of sites that showed the strongest signal of coevolution through evolutionary times (as measured through the Frobenius norm of the couplings of Potts model) were the ones that deviated the most from the macroscopic model. These idiosyncratic epistatic interactions seem, therefore, to generate in the long-term some co-evolution patterns between pairs of sites that can be captured by models trained on MSA.

The fact that the experimental epistasis we characterized as either global or idiosyncratic can both be recovered to some extent from the analysis of distant homologs is telling us that the molecular determinants of epistasis are long-lasting. It suggests that the persistence of the underlying mechanistic selective pressures has been long and strong enough to shape the long-term evolution of the protein family. Despite the wide-spread level of epistasis we recovered in our data, these observations reject a model in which epistatic interactions are fully volatile and change quickly with protein sequence, as suggested for instance in the NK model^52^. Our data suggest a rather smooth and consistent protein mutational landscape, as some experimental data comparing homologs^26^, the impact on stability of mutations in human protein domains^22^, or the conservations of context dependency over long evolutionary time scales suggest^53^. This consistency offers the hope that protein landscape properties could be tractable and extrapolated from one homolog to another using combinations of mutational scans and in-depth MSA analysis^54^.

Surprisingly, in this study, fitness and MIC measurements yielded remarkably similar results. This outcome was unexpected for several reasons. First, fitness estimates derived from competition assays are significantly more accurate than MIC estimates, a critical consideration for reliably assessing epistasis. Second, fitness measured at a specific concentration exhibits a much narrower dynamic range compared to MIC; mutants that reduce fitness by half are indistinguishable from non-viable mutants, whereas MIC can capture over a 32-fold reduction in efficiency. Third, it is fitness, rather than an integrated functional metric like MIC, that drives the evolutionary trajectories of protein sequences. Consequently, one might have anticipated that the MSA would provide greater insights into fitness than MIC. Conversely, as the relevant concentration for evolution remains an open question, one could have argued that MIC, which somehow integrates mutation effects at a broader scale, could have been more relevant.

Our analysis indicates that the effects observed in the α-helix under study appear to be governed primarily by an integrated phenotype, specifically protein stability. Through this integrated phenotype, mutations may interact, and their effects and interactions can subtly influence both MIC and fitness measurements. Despite many mutations being highly destabilizing, their fitness effects can be partially offset when combined with stabilizing mutations, as previously described^55^. This compensation enables presumably the exploration of complex sequence pathways over evolutionary timescales and allows coupling effects to manifest in the MSA.

## Methods

### Strains and plasmids

#### Strains

*E. coli* strains used in this study: **XL1-Blue** (Agilent, Santa Clara, CA) of genotype recA1 endA1 gyrA96 thi-1 hsdR17 supE44 relA1 lac [F’ proAB lacI1qZΔM15 Tn10 (Tetr)]; **CJ236** (new England biolabs) of genotype FΔ(HindIII)::cat (Tra+ Pil+ CamR)/ ung-1 relA1 dut-1 thi-1 spoT1 mcrA; **DH5α** (Invitrogen) of genotype F– Φ80lacZΔM15 Δ(lacZYA-argF) U169 recA1 endA1 hsdR17 (rK–, mK + ) phoA supE44 λ– thi-1 gyrA96 relA1; **Dh10b** Electromax (ThermoFisher Scientific) of genotype F^-^*mcr*A Δ(*mrr*-*hsd*RMS-*mcr*BC) Φ80*lac*ZΔM15 Δ*lac*X74 *rec*A1 *end*A1 *ara*D139Δ(*ara*, *leu*)7697 *gal*U *gal*K λ^-^*rps*L *nup*G

#### Plasmids

Phagemid **pSkunk3-TEM-1** was obtained graciously from Elad Firnberg and Marc Ostermeier. The **pSkunk-TEM-helix** was created by inserting a *Nco*I restriction site 2 bases before the beginning of alpha helix to mutagenize using single-step Pfunkel mutagenesis^56^. We also inserted *Xho*I and *Not*I restriction sites, which surround the streptomycin/spectinomycin (Sm/Spec) resistance gene. Plasmid pKD3 was used to amplify the cat gene encoding chloramphenicol (Cm) resistance. The **pSkunk-TEM-helix-Cm** was created by swapping the Sm/Spec resistance gene with a Cm resistance gene and adding a DNA barcode of 20 degenerate nucleotides.

### Construction of a library of barcoded mutants

In brief, the sequence of TEM-1 was mutagenized using a previously published phagemid^56^ that was slightly modified. This phagemid allows high throughput mutagenesis to be performed, from a phage mediated single stranded amplification, and the synthesis of the other strand using a pool of mutated oligonucleotides. For our purposes, these oligonucleotides each carry two degenerate NNS codons (N is either A, T, G, or C; S is either G or C) in the alpha helix of interest. A collection of 150,000 mutants was made with this protocol. The mutants were then combined through Gibson assembly^57^ to a genetic barcode of sequence NNNNNATNNNNNATNNNNNATNNNNN flanking a gene providing resistance to the antibiotic chloramphenicol. Two million barcoded mutants were recovered. Here are the details of the mutagenesis protocol.

#### ssDNA production

Briefly, the phagemid pSkunk-TEM-helix was transformed into *E. coli* CJ236 cells, which were selected on LB agar supplemented with spectinomycin (50 µg/mL), chloramphenicol (15 µg/mL), and deoxythymidine (125 µg/mL) and incubated overnight at 30 °C. A single colony was used to inoculate LB medium containing the same antibiotics and grown overnight at 30 °C with shaking. Cell density was estimated from OD600 using a conversion factor of 2 × 10⁸ CFU/mL per OD unit. For phage production, 2 × 10⁷ CFU were inoculated into 2 mL of TBG medium supplemented with spectinomycin and infected with R408 helper phage at a multiplicity of infection (MOI) of 5. Cultures were incubated for 6 h at 37 °C with shaking. Cells were pelleted by centrifugation, and phage particles were precipitated from the supernatant using polyethylene glycol (PEG)/NaCl. After centrifugation (26,200 g for 1 h at 4 °C), the phage pellet was resuspended in PBS, and dU-containing pSkunk-TEM-helix single-stranded DNA was purified using the QIAprep Spin M13 kit (Qiagen) according to the manufacturer’s instructions. ssDNA concentration was quantified using the Qubit® ssDNA Assay Kit (ThermoFisher Scientific).

#### Single step Pfunkel mutagenesis

The sequence of TEM-1 was mutagenized using pSkunk-TEM-helix ssDNA as a matrice. The synthesis of the other strand used a pool of mutated oligonucleotides. For our purposes, these oligonucleotides each carry two degenerate NNS codons (N is either A, T, G, or C; S is either G or C) in the alpha helix of interest (Supplementary Table 19) and were first phosphorylated using T4 polynucleotide kinase (PNK) at 37 °C for 1 h, followed by enzyme inactivation at 65 °C for 20 min.

Mutagenesis was performed with 1 µg of dU-containing pSkunk-TEM-helix ssDNA used as template in a total volume of 100 µl containing 1× PfuTurbo Cx Hotstart DNA polymerase buffer, 10 mM DTT, 0.5 mM NAD⁺, 0.2 mM dNTPs, 1 µL of the kinase reaction (pool of phosphorylated primers), 2.5 U PfuTurbo Cx Hotstart DNA polymerase, and 200 cohesive end units of Taq DNA ligase. Thermal cycling consisted of 95 °C for 3 min, 55 °C for 90 s, 68 °C for 20 min, and 45 °C for 15 min. Subsequently, 3.8 pmol of oligonucleotide P320 was added, followed by one additional cycle (95 °C for 30 s, 55 °C for 45 s, 68 °C for 20 min, and 45 °C for 15 min).

To remove the template strand, 10 U uracil-DNA glycosylase (UDG) and 30 U exonuclease III were added, and the reaction was incubated at 37 °C for 1 h, followed by heat inactivation at 70 °C for 20 min. We used the innuPREP PCRpure Kit (Analytik Jena) to purify DNA and eluted in 15 µl of distilled DNAse/RNAse free water. 2 µl were then electroporated in 20 µl of DH5α electrocompetent cells and then incubated with 500 µl of LB media for 1 hour at 37 °C with shaking at 250 rpm. The transformation was plated on LB agar with 50 µg/ml streptomycin and then incubated overnight at 37 °C. A pool of 150,000 colonies was scraped from the LB agar plates (245 mm × 245 mm, Greiner bio-one) in LB broth and frozen at −80 °C in LB/glycerol 40%. After pooling all colonies together, plasmids were extracted from an aliquot using plasmid miniprep (Qiagen, Valencia, CA), forming the library of mutants.

#### Mutant barcoding

The mutants were then combined through Gibson assembly to a genetic barcode flanking a gene providing resistance to the antibiotic chloramphenicol. For that, 10 µg of plasmid extraction of the library of mutants was digested with NotI, XhoI, and NcoI (buffer 3.1), in 500 μl total reaction volume (New England Biolabs), gel extracted (band of 3350 bp) using Qiagen Gel Extraction kit, and then also cleaned a 2nd time with Qiagen PCR Purification kit. The final concentration was 25 ng/µl.

pKD3 was used as template for PCR amplification of *cat* using specific primers that also contained overlapping regions of pSkunk-TEM-helix (for subsequent Gibson Assembly). The forward primer also contained a non-overlapping region with a DNA barcode consisting of 20 degenerated nucleotides of sequence NNNNNATNNNNNATNNNNNATNNNNN (Supplementary Table 19). Phusion® High-Fidelity DNA Polymerase (New England Biolabs) was used with reaction cycling conditions: 98 °C for 30 s, followed by 35 cycles of 98 °C for 10 s, and 62 °C for 30 s, 72 °C for 15 s, and a final extension at 72 °C for 2 min.

The plasmid pSkunk-TEM-helix-Cm was created by switching the spectinomycin/streptomycin resistance with the Cm resistance cassette amplified previously using Gibson Assembly (New England Biolabs). This allows the integration of the DNA barcode. Gibson reaction was carried out with 3 µl of 25 ng/ul of plasmid fragment and 1.3 µl of 88 ng/µl of barcode-CmR-amplicon (1:5 molar ratio backbone:insert), in a total of 20 µl reaction mix and incubated at 50 °C for 1 h. The total volume of Gibson reaction was dialyzed with water for 30 min and 4 µl was electroporated in 20 µl of Dh10b Electromax competent cells that were then incubated in 500 µl of LB media for 1 h at 37 °C with shaking at 250 rpm. The transformants were plated on LB agar with 25 µg/ml chloramphenicol and then incubated overnight at 37 °C. A pool of about 2.10^6^ colonies was scraped from the LB agar plates (245 mm × 245 mm, Greiner bio-one) in LB broth and frozen at −80 °C in LB/glycerol 40%.

### Coupling barcode sequences to mutant sequence

To find for each barcode the mutations in the alpha helix it is linked to, two independent PCRs (including one in emulsion) were done with one end of the product corresponding to the barcode and the other end to the alpha helix sequence. For that, a two-step PCR method was used to amplify the corresponding part of the gene, including the alpha helix sequence on 5’ part and barcode sequence on 3’ extremity, and to add the Illumina sequencing adapter and multiplex barcode sequences. In detail, plasmid DNA concentration was determined using qubit fluorometric quantification (ThermoFisher Scientific) and normalized to 2.5 ng/µl. Three reactions using 1 ng of DNA were used for the 1st PCR using specific primers and allowed the attachment of an adaptor that is necessary for the 2nd PCR. Between specific primers and adaptors, 6 degenerate nucleotides were inserted in order to increase the diversity of DNA to facilitate MiSeq clustering (by improving crosstalk and phasing calculations) (Supplementary Table 19). In order to decrease chimera formation, and to reduce further amplification bias that arises during PCR, a second PCR was made with an emulsion-PCR protocol (Micellula, following the manufacturer’s guidelines). The emulsion PCR allows compartmentalization of individual DNA molecules within micelles, enabling isolated amplification reactions by physically separating templates in a single tube. The process begins with the preparation of a water phase containing the PCR reaction components (50 µl of Kapa Hifi Hotstart Ready Mix PCR Kit polymerase (Kapa Biosystems) added with 1 µl of BSA and 1 ng of DNA) and an oil surfactant mixture to form a stable emulsion (300 µl of Oil Surfactant Mixture (precooled at 4 °C) added with 50 µl of the water-phase. Following vortexing to create the micelle compartments, thermal cycling is performed under 95 °C for 30 s, followed by 12 cycles of 95 °C for 10 s, 55 °C for 30 s, 68 °C for 30 s, and a final extension at 68 °C for 5 min. After amplification, the emulsion is broken using 2-butanol and vortexing, and the DNA is purified via spin-column chromatography following the supplier’s recommendations. After gel purification using Qiagen gel extraction kit (Valencia, CA), DNA was quantified using Qubit fluorometric quantification, and DNA concentration was normalized. The 2nd PCR was performed using 0.3 ng of DNA using primers commercialized by Illumina in the Nextera Index Kit, allowing the dual indexing. The reaction cycling conditions were the same as previously, but only 11 cycles were performed using Kapa Hifi Hotstart Ready Mix PCR Kit polymerase (Kapa Biosystems). After gel purification with Qiagen gel extraction kit (Valencia, CA), quantification using qPCR kapa Hifi Hotstart (Kapa Biosystems) on a Light cycler 480 Roche was performed with reaction cycling conditions of 95 °C for 5 min, followed by 35 cycles of 95 °C for 30 s and 60 °C for 45 s as specified by Kapa Biosystems. This library, corresponding to the first time-point (T0), was diluted to 12 pM and loaded on the MiSeq with a mix of 10% PhiX DNA (PhiX Control v3, illumina). Three MiSeq V3 2 × 75 bp paired-end runs (Illumina technology) were performed for this part, resulting in a total of > 40 M reads, for an expected ~20x coverage of barcode diversity. The paired-end reads are non-overlapping, with the alpha helix sequence on Read 1 and the barcode sequence on Read 2.

### Barcode-mutant association

The following steps were performed using the Mothur software package^58^ (https://www.mothur.org/). Raw reads from all sequencing runs were pooled together and quality-filtered by size (>69 bases), number of uncalled bases (<3 Ns), and length of longest homopolymer stretch, an indicator of overall read quality (<13 bases). Alpha helix and barcode sequences were extracted from Read 1 and Read 2, respectively, after alignment to the reference sequences (Needleman global alignment). Reads for which either the alpha helix or barcode region contained insertions or did not generate a full alignment with the reference were discarded. The Mothur precluster algorithm was then used to cluster barcode sequences differing by a Hamming distance of 1, with the aim of correcting for PCR and sequencing errors (the potential barcode diversity is so high that the presence of immediately neighboring sequences is very likely due to these errors). The algorithm uses sequence abundance to decide the “true” (majority) sequence for each cluster, and to decide where a sequence clusters if it has >1 immediate neighbor. After de-gapping and re-clustering barcode sequences to account for any alignment ambiguities resulting from small deletions, barcode clusters were used to build a dictionary assigning each “true” barcode sequence to an alpha helix sequence. Due to the high rate of PCR-derived recombination observed (caused by the long homologous region between the barcode region and alpha helix sequence, and resulting in molecules with swapped barcodes), a haplotype-based strategy was used for this step rather than one in which each nucleotide is considered independently. This is because the small number of mutations present in each mutant means that, at any particular position, the majority of molecules will possess the WT base, and so a high recombination rate can result in consensus alpha helix sequences in which mutant bases are assigned as WT. The efficiency of this strategy was ensured by the short length of the mutagenized region and high quality of the reads, meaning that most reads did not contain a single error in the regions of interest and so were not wasted. Briefly, for each barcode cluster (consisting of reads whose barcode sequences are identical to or the immediate neighbor of the inferred “true” barcode sequence), the paired alpha helix sequences were fetched; the number of occurrences of each resulting alpha helix sequence was tabulated; if the cluster contains more than 2 reads in total, the most abundant alpha helix sequence is ≥5x more abundant than the second-most abundant alpha helix sequence, and the most abundant alpha helix sequence contains no Ns, then the most abundant alpha helix sequence is assigned to the “true” barcode sequence for that cluster (else the cluster is discarded).

To prevent wrong association of some barcodes to diverse alpha helix sequences due to recombination that may occur during the PCR, we excluded from the analysis barcodes for which the second most frequent alpha helix sequence had a frequency higher than 20%.

### Selection experiment

To infer fitness of these mutants, a competition experiment was performed. The collection was grown in 100 ml of MH broth in flasks to an Optical Density (OD) of 0.4 in the absence of amoxicillin, and subsequently diluted 32-fold in 100 ml of MH broth, this time supplemented with 8 g/l amoxicillin. The optical density was followed through time, and as soon as OD reached 0.2, a 32-fold dilution was performed in fresh media with the same concentration of antibiotics. Up to 6 cycles were performed, corresponding to about 30 generations. At each dilution, samples were taken to purify the plasmid and sequence the barcode.

### Barcoding sequencing

Given that barcodes are now associated to mutations, to track mutant frequencies in the course of the selection experiment, we only need the sequencing of barcodes at the different time-points (T0, T1, T2, T4, and T6). For this, a similar protocol was carried out using oligonucleotides that surround the barcode region; employing the same 2-step PCR-based method and similar conditions Supplementary Table 19). In this case, the 6 degenerate nucleotides inserted on either side of the barcode region during the 1st PCR also allowed us to remove PCR duplicates arising from the 2nd PCR. All libraries corresponding to the different evolution time-points were quantified using a qPCR-based method (Integragen) and pooled in equal molar quantity. They were then sequenced on a HiSeq4000 with a 2 × 100 bp paired-end kit (Illumina technology) by Integragen society, to give overlapping reads of the barcode region. The run resulted in ~300 M raw paired-end reads, and so ~27 M for each of the 10 time-points/conditions (duplicate). This gives a barcode coverage of ~14x for each time-point/condition.

### Barcode counting

The following steps were performed using the Mothur software package^58^. Demultiplexed forward and reverse reads were joined into contigs using Mothur’s make.contigs command with the default parameters, which takes into account the Phred score to assign (or not) a base when there is disagreement between forward and reverse reads. Contigs were then quality-filtered by size (<151 bp, as longer contigs imply forward and reverse reads could not be properly overlapped), number of uncalled bases (no Ns), and length of longest homopolymer stretch, an indicator of overall read quality (<13 bases). To remove the majority of PCR duplicates arising from the 2nd PCR (made possible by the 6 degenerate nucleotides introduced on each side of the barcode during the 1st PCR), if a particular contig was present more than once, only one copy was kept. Barcode sequences were then extracted after aligning full contigs to the reference sequence (Needleman global alignment). Reads containing insertions or not generating a full alignment with the reference were discarded. Next, the Mothur precluster algorithm was used to cluster barcode sequences differing by a Hamming distance of 1, with the aim of correcting for PCR and sequencing errors, as described above for the barcode-mutant association. After de-gapping and re-clustering barcode sequences to account for any alignment ambiguities resulting from small deletions, the number of occurrences of each “true” barcode was tabulated across all time-points/conditions. Finally, a custom R script was used to merge the barcode-mutant dictionary generated above with the barcode counts table.

Based on previous work, in which we found no clear effect of synonymous mutations, we combined all synonymous mutations into a single allele.

### Quality control of barcodes

Multiple Barcodes were associated to the different genotypes. Several processes may lead to variability in the signal provided by the different barcodes. First, though we used some correction and some emulsion PCR to try to correct that bias, some recombination may occur during the PCR between the part of the protein and the barcode and escape our detection procedures. Hence, a Barcode may appear to be associated to the focal genotype, but may indeed correspond to an alternative genotype. Even if a barcode is associated properly to its alpha-helix genotype, we have not sequenced the whole protein. Consequently, an undetected mutation may affect the protein elsewhere and result in a modified behavior of that barcode. To limit the effect of these outliers, which are often barcodes associated with loss of function or maximal fitness, we first did a screen to filter outlier barcodes.

For that purpose, we computed the change in the focal genotype to wild-type genotype frequency over the first cycle of evolution (T0 to T1), using the sum of all barcodes linked the focal genotype.

11 $${K}_{j}=\left(\frac{{\sum }_{i}{{BC}}_{{ij}}^{1}}{{{Wt}}^{1}}\frac{{{Wt}}^{0}}{{\sum }_{i}{{BC}}_{{ij}}^{0}}\right)$$

in which ${{BC}}_{{ij}}^{1}$ is the number of reads matching the jth barcode associated to genotype i at time 1, and ${{Wt}}^{1}$ the number of reads matching barcodes associated to wild type sequence. The value of K corresponds to an estimate of fitness over one cycle. Then, for each individual barcode, we can compute based on ${{BC}}_{{ij}}^{0}$ the estimated number of reads expected at T1. If the barcode is following the overall trend we expect

12 $${K}_{{ij}}=\left(\frac{{{BC}}_{{ij}}^{1}}{{{Wt}}^{1}}\frac{{{Wt}}^{0}}{{{BC}}_{{ij}}^{0}}\right)={K}_{j}$$

We expect, therefore, ${{BC}}_{{ij}}^{1}$ to be distributed with a Poisson law of parameter $\frac{{\sum }_{i}{{BC}}_{{ij}}^{1}}{{\sum }_{i}{{BC}}_{{ij}}^{0}}{{BC}}_{{ij}}^{0}$.

All barcodes, with a *p*-value lower than 10^−5^ were assumed to reject that model and to be the result of some of the artifacts previously mentioned. They were discarded, and this selective process was rerun once to be sure to eliminate all outliers. Reads matching all the remaining barcodes were then combined to estimate fitness. Furthermore, barcodes with less than 10 counts for the combined time T0 and T1 were excluded, as well as mutants with less than 4 barcodes.

### Inference of log-fitness

For a given mutant i, we consider that the total population of plasmids ${N}_{i}$ carrying this mutant follows an exponential growth

13 $${N}_{i}(t+1)={W}_{i}\times {N}_{i}(t)$$

where ${W}_{i}$ denotes the absolute fitness of the mutant *i*. However, we do not have access to the total population over time but to some measurements of the population ${\left\{{\hat{N}}_{i}\left({T}_{k}\right)\right\}}_{k}$ at different times ${T}_{k}$ sampled by a DNA sequencer. Consequently, we construct an inference procedure to estimate its absolute fitness ${W}_{i}$ knowing the measurements of the population at different times. The probability of ${\left\{{\hat{N}}_{i}\left({T}_{k}\right)\right\}}_{k}$ at different times ${T}_{k}$ knowing ${W}_{i}$ can be written as

14 $$	 P\left({\left\{{\hat{N}}_{i}\left({T}_{k}\right)\right\}}_{k}|{W}_{i}\right) \\ 	={\sum }_{{N}_{i}(0)\ge 0}{\prod }_{k}\left(\begin{array}{c}{d}^{k}{W}_{i}^{{T}_{k}}{N}_{i}(0)\\ {\hat{N}}_{i}\left({T}_{k}\right)\end{array}\right){{{p}_{k}}^{{\hat{N}}_{i}\left({T}_{k}\right)}\left(1-{p}_{k}\right)}^{{d}^{k}{W}_{i}^{{T}_{k}}{N}_{i}(0)-{\hat{N}}_{i}\left({T}_{k}\right)}$$

where $d=\frac{1}{32}$ denotes the dilution ratio and ${p}_{k}$ the sampling rate of the DNA sequencer at time ${T}_{k}$. The summation is carried out over the unknown initial population size ${N}_{i}(0)$. Assuming a flat prior, the absolute fitness W_i_ is estimated by maximizing the probability *P* above. Further information about the definition and maximization of *P*, as well as on the estimation of the ${p}_{k}$’s can be found in Supplementary Note 1.

### Computing MIC

For MIC determination, we used multiple approaches. First, the read counts were turned to absolute counts by multiplying them by the survival counts at each of the concentrations averaged over four independent replicates (Supplementary Table 20). Then the relation between counts and concentration was smoothed with a local polynomial regression fitting (loess function in R) with a span of 0.75. As some of the counts are low this smoothing decreases the impact of outlier counts. We then used 3 independent measures of MIC and combined them in a 4th. All of them rely on log2 transforms of concentrations. The first one is the extrapolation of the concentration at which the counts have decreased by 75% compared to the no antibiotics counts. We refer to this measure as MIC_EC75_. The second estimate of MIC is the area under the curve. We refer to this measure as MIC_Area_. The third one is a relying on a moment matching approach, which we refer to it as MIC_MMA_. This approach uses the smoothed connection between counts and concentration to fit a step function that has same mean and variance than the read counts. In detail for mutant *i*, the variance *V*_i_ and mean *M*_i_ of the smoothed number of reads, x_i_, were used in the following formula to compute MIC_MMA_:

15 $${{MIC}}_{{MMA}{{\_}}i}=\frac{12}{1+\frac{{V}_{i}}{{\left({M}_{i}\right)}^{2}}}$$

Where 12 is the number of concentrations used. The Supplementary Fig. S1 illustrate these three measures of MIC for a given mutant. Pearson’s correlations between these estimates of MIC between the two replicates were 0.949 for MIC_Area_, 0.992 for MIC_MMA_, and 0.991 for MIC_EC75_, Spearman correlations were 0.916 for MIC_Area_, 0.914 for MIC_MMA_, and 0.924 for MIC_EC75_. Within replicates, the different estimates correlated highly as well: Pearson’ correlations MIC_Area_/MIC_MMA_ > 0.97, MIC_Area_/MIC_EC75_ > 0.97, MIC_MMA_/MIC_EC75_ > 0.997, and Spearman’s correlations MIC_Area_/MIC_MMA_ > 0.995, MIC_Area_/MIC_EC75_ > 0.97, MIC_MMA_/MIC_EC75_ > 0.97. Because MIC_Area_ had lower reproducibility, we did not consider it later on.

We then combined MIC_MMA_ and MIC_EC75_ through a principal component analysis based on these two measures independently for the two replicates (scaling each component). In both cases, the first component explained more than 99.9% of the variance. The value along that axes was used as a fourth value of MIC, MIC_Combined_. The correlation between replicates was 0.992 and 0.911 for Pearsons of Spearman’s correlation coefficient, respectively.

For further studies and notably measures of epistasis, the values of these MIC estimates were computed as difference to the value of the wild-type. We then used mutants with stop codons or frameshifts to established the score associated with nonfunctioning enzyme. The threshold value defining functionality, MIC_f_ was set to the mean effect of these nonsense mutants plus 1.96 standard deviation.

### Selection of mutants for epistasis

To have high resolution on epistasis, we calculate it only for double mutants that have log-fitness greater than −0.6 and whose two associated single mutants have log-fitness greater than −0.6. For MIC, epistasis between mutants A and B was computed as ${\varepsilon }_{{AB}}={{MIC}}_{{AB}}-{{MIC}}_{A}-{{MIC}}_{B}$. Epistasis could only be quantitatively measured if all 3 MICs in the previous equation were higher than the threshold MIC_f_. We could find that epistasis was present but could only be bound when ${{MIC}}_{A}+{{MIC}}_{B} < {{MIC}}_{f}$ and ${{MIC}}_{{AB}} > {{MIC}}_{f}$, which means ${\varepsilon }_{{AB}} > {{MIC}}_{{AB}}-{{MIC}}_{f}$ or when ${{MIC}}_{{AB}} < {{MIC}}_{f}$ and ${{MIC}}_{A}+{{MIC}}_{B} > {{MIC}}_{f}$, which means ${\varepsilon }_{{AB}} < {{MIC}}_{f}-{{MIC}}_{A}-{{MIC}}_{B}$. These cases, however, not included in the statistical approaches.

### Inference of $\Delta \Delta G$ on single and double mutants

We denote as ${w}_{i}^{a}$ the relative fitness of the mutant that carries amino acid $a$ at site $i$ of the $\alpha$-helix. We denote as ${w}_{i,j}^{a,b}$ the relative fitness of the mutant with amino acids $a$ and $b$ at, respectively, sites $i$ and $j$.

The stability model reads:

16 $${\mathrm{ln}}\left({w}_{i}^{a}\right)={\mathrm{ln}}\left(1+{e}^{\frac{\Delta {G}_{0}}{{RT}}}\right)-{\mathrm{ln}}\left(1+{e}^{\frac{\Delta {G}_{0}+\Delta \Delta {G}_{i}^{a}}{{RT}}}\right)$$

17 $${\mathrm{ln}}\left({w}_{i,j}^{a,b}\right)={\mathrm{ln}}\left(1+{e}^{\frac{\Delta {G}_{0}}{{RT}}}\right)-{\mathrm{ln}}\left(1+{e}^{\frac{\Delta {G}_{0}+\Delta \Delta {G}_{i}^{a}+\Delta \Delta {G}_{j}^{b}}{{RT}}}\right)$$

To fit the parameters of this model, we assign every single mutant a free-energy value, $\Delta \Delta G$, reflecting the impact of the mutant on the whole protein stability, as well as an overall free energy scale, $\Delta {G}_{0}$. Though measures of $\Delta {G}_{0}$ have been done in vitro, the cellular environment in which the mutants are evaluated could substantially affect the value. We, therefore, also infer $\Delta {G}_{0}$ from our fitness data. Ideally, the estimated log-fitness is directly connected to $\Delta \Delta G$ for the single mutants by inverting Eq. 12. However, this procedure has limited accuracy. For the inference of the $\Delta \Delta G$, we keep single mutants with log-fitness greater than $-0.6$ only. For each pair of previously chosen single mutants, the associated double mutant is kept if it exists. Its relative log-fitness is thresholded at $-0.6$. The stability model is itself thresholded at $-0.6$ during the inference.

As the model is over-constrained, we introduce a regularization procedure explained in $\Delta \Delta {G}_{i}^{a}$ and $\Delta {G}_{0}$ are estimated by minimizing the following cost function, which corresponds to a robust nonlinear regression

18 $$C\left(\Delta {G}_{0},\left\{\Delta \Delta {G}_{i}^{a}\right\}\right)=\frac{1}{2}{\sum }_{i}{\alpha }_{i}{T}^{2}\Phi \left(\frac{{r}_{i}^{2}}{{T}^{2}}\right)$$

Where ${r}_{i}$ is the residual

19 $${r}_{i}=\frac{{\mathrm{ln}}\left({W}_{i}\right)-{\mathrm{ln}}\left({\hat{W}}_{i}\right)}{{\sigma }_{{\mathrm{ln}}\left({W}_{i}\right)}}$$

with ${\mathrm{ln}}\left({W}_{i}\right)$ the log-fitness of the mutant, ${\mathrm{ln}}\left({\hat{W}}_{i}\right)$ the theoretical log-fitness of the mutant given by the two-state model (Eq. 3) and ${\sigma }_{{\mathrm{ln}}\left({W}_{i}\right)}$ the standard deviation of the log-fitness inferred with our inference procedure (Supplementary Note 4).

If *α*_*i*_ *=* 1 and Φ(*x*) = *x*, the cost function corresponds to the canonical least-squares estimation. However, as we perform the inference on single and double mutants, single mutants are underrepresented compared to double mutants, which are much more numerous. We compensate for this effect by choosing adequate statistical weights ${\alpha }_{i}$: for the double mutants, ${\alpha }_{i}=2$; for the single mutants, ${\alpha }_{i}$ is equal to the number of double mutants with this single mutation.

To penalize the strong outliers found in our data, we used $\Phi \left(x\right)=\arctan (x)$ as a loss. The parameter T is a threshold that controls the importance of the regularization of the outliers and is chosen such that 30% of the mutations are considered as outliers. The results are consistent for a wide range of thresholds T (from *T* = 20 to *T* = 100), penalizing only the strong outliers. For the parameters shown in the paper, *T* = 50.

### Selection of mutants for $\Delta \Delta G$ and $\Delta {G}_{0}$ inference

For the inference of $\Delta \Delta G$ and $\Delta {G}_{0}$ from the single mutational scan, we kept all single mutants with a log-fitness greater than −0.6 or higher than the functionality threshold for MIC measurements. For the inference of the same parameters from the single and double mutants, we add to the fit all the double mutations for which the single mutants are kept; but, if their log-fitness (MIC) is smaller than -0.6 (functionality threshold), we threshold it to −0.6 (functionality threshold).

### Inference of independent model and Potts model

All models are trained by maximizing the log-likelihood of MSA built on homologs of class A β-lactamases cleaned by hand^35,36^ enriched on SwissProt and TrEMBL^37^, with a total of *B* = 8749 sequences with length *L* = 253. Each sequence is reweighted according to the classical reweighting scheme^38^, with a threshold equal to 0.2, leading to an effective number of sequences ${B}_{{eff}}=2480$. The Potts model was inferred through pseudolikelihood maximization^59,60^ with ${L}_{2}$ regularization (for the couplings, ${\gamma }_{J}=\frac{L}{{B}_{{eff}}}$, and for the fields, ${\gamma }_{h}=\frac{0.1}{{B}_{{eff}}}$) and color compression^61^, with a threshold ${f}_{0}=0$.

### Codes

R software^62^ as well a Jupyter^63^ notebooks were used for data manipulation, inferences, models and figures.

### Reporting summary

Further information on research design is available in the Nature Portfolio Reporting Summary linked to this article.

## Supplementary information

Supplementary Information

Reporting Summary

## Source data

Source data

## Data Availability

The sequencing data generated in this study have been deposited in the European Nucleotide Archive database under accession PRJEB10446. Source data are provided with this paper.

## Code availability

All codes to process the raw data, compute barcode coupling and frequencies, infer fitness, as well as ∆∆Gs as well as to reproduce all figures are accessible at Zenodo with 10.5281/zenodo.18457561.
