# Supplementary material for: Origins and breadth of pairwise epistasis in an α-helix of β-lactamase TEM-1
Source: Nat Commun. 2026 Mar 17;17:4083. doi: 10.1038/s41467-026-70627-5 (PMC13144301; doi:10.1038/s41467-026-70627-5)
Supplement: Supplementary file 1 — Supplementary Information [file 41467_2026_70627_MOESM1_ESM.pdf]

Supplementary Tables

Supplementary Table 1: Fraction of lethal mutants among single and double mutants according to the different metrics and replicates.

Measure	Number of double mutants	Fraction of lethals among single mutants	Fraction of lethals among double mutants	Fraction of double mutants including at least lethal single mutant	Fraction of truly non-lethal double mutants including at least lethal single mutant	Fraction of truly non-lethal double mutants composed of two lethal single mutants (>5/6 of lethal threshold)
MIC_{mma} replicate 1	14092	0.1483	0.4612	0.2757	0.0049	0.0005
MIC_{mma} replicate 2	14092	0.1340	0.4478	0.2528	0.0042	0.0000
MIC_{75} replicate 1	14092	0.1388	0.4604	0.2622	0.0038	0.0005
MIC_{75} replicate 2	14092	0.1340	0.4505	0.2528	0.0042	0.0000
$MIC_{combined}$ replicate 1	14092	0.1388	0.4592	0.2622	0.0038	0.0005
$MIC_{combined}$ replicate 2	14092	0.1340	0.4479	0.2528	0.0042	0.0000
Log Fitness replicate 1	14092	0.4689	0.7777	0.7100	0.0095	0.0000
Log Fitness replicate 2	14092	0.4737	0.7768	0.7158	0.0104	0.0000

Supplementary Table 2: Correlations between MIC measurements and the associated Log Fitness estimates (same replicate) for single and double mutants. Pearson's r and Spearman's ρ are reported.

Measure	Single Mutants (Pearson)	Single Mutants (Spearman)	Double Mutants (Pearson)	Double Mutants (Spearman)
MIC_{mma} replicate 1	0.8866	0.9394	0.8702	0.7855
MIC_{mma} replicate 2	0.9121	0.9732	0.8878	0.8023

Measure	Single Mutants (Pearson)	Single Mutants (Spearman)	Double Mutants (Pearson)	Double Mutants (Spearman)
MIC_{75} replicate 1	0.8813	0.9417	0.8661	0.7832
MIC_{75} replicate 2	0.9039	0.9721	0.8805	0.8031
$MIC_{combined}$ replicate 1	0.8841	0.9400	0.8685	0.7849
$MIC_{combined}$ replicate 2	0.9083	0.9729	0.8845	0.8012

Supplementary Table 3: Correlations between epistasis measurements among replicates for the different metrics. Pearson's r and Spearman's ρ are reported.

	MIC_{mma}	MIC_{75}	$MIC_{combined}$	Log Fitness
Epistasis between replicates (Pearson)	0.9141	0.9043	0.9129	0.9951
Epistasis between replicates (Spearman)	0.8844	0.8840	0.8859	0.9718

Supplementary Table 4: Correlations between epistasis measurements among replicates for the different metrics. Pearson's r and Spearman's ρ are reported.

	MIC_{mma} replicate 1	MIC_{mma} replicate 2	MIC_{75} replicate 1	MIC_{75} replicate 2	$MIC_{combined}$ replicate 1	MICcombined replicate 2
Log Fitness Epsitasis replicate 1 (Pearson)	0.5689	0.6061	0.5369	0.5709	0.5579	0.5960
Log Fitness Epsitasis replicate 1 (Spearman)	0.5435	0.6078	0.5436	0.6209	0.5463	0.6171
Log Fitness Epsitasis replicate 2 (Pearson)	0.5716	0.6119	0.5376	0.5759	0.5585	0.6022
Log Fitness Epsitasis replicate 2 (Spearman)	0.5445	0.6091	0.5431	0.6203	0.5467	0.6171

Supplementary Table 5: Correlations between epistasis measurements among the different MIC metrics and replicates. Spearman's ρ are reported.

	MIC_{mma} replicate 1	MIC_{mma} replicate 2	MIC_{75} replicate 1	MIC_{75} replicate 2	$MIC_{combined}$ replicate 1	$MIC_{combined}$ replicate 2
MIC_{mma} replicate 1	1.0000	0.9141	0.9907	0.9001	0.9976	0.9107
MIC_{mma} replicate 2	0.9141	1.0000	0.9057	0.9869	0.9127	0.9966
MIC_{75} replicate 1	0.9907	0.9057	1.0000	0.9043	0.9978	0.9087
MIC_{75} replicate 2	0.9001	0.9869	0.9043	1.0000	0.9052	0.9969
$MIC_{combined}$ replicate 1	0.9976	0.9127	0.9978	0.9052	1.0000	0.9129
$MIC_{combined}$ replicate 2	0.9107	0.9966	0.9087	0.9969	0.9129	1.0000

Supplementary Table 6: Categorization of the 209 single mutations as lethal, destabilizing, neutral or stabilizing based on their effect when combined with other mutations. Lethal refer to mutation that are lethal when combined to any other mutants but two. Destabilizing mutations are mutations that show a positive correlation with the background fitness (controlling for lethal combinations) their effect is worth when combined to a deleterious background. Stabilizing are the ones showing a negative correlation with background fitness. Their effect is more positive when they are associated to deleterious backgrounds and Neutral are the mutations showing no clear pattern with background fitness, mutation that tend to have almost no fitness effect.

Measure	Lethal	Destabilising	Neutral	Stabilising
MIC_{mma} replicate 1	21	158	19	11
MIC_{mma} replicate 2	19	161	19	10
MIC_{75} replicate 1	23	156	19	11
MIC_{75} replicate 2	21	158	20	10
$MIC_{combined}$ replicate 1	21	158	19	11
$MIC_{combined}$ replicate 2	21	159	19	10
Log Fitness replicate 1	73	116	9	11
Log Fitness replicate 2	72	117	8	12

Supplementary Table 7: Correlation between the inferred values of ΔΔG for the non-lethal single mutants, across metrics and replicates. Spearman's ρ are reported.

	MIC_{mma} replicate 1	MIC_{mma} replicate 2	MIC_{75} replicate 1	MIC_{75} replicate 2	$MIC_{combined}$ replicate 1	$MIC_{combined}$ replicate 2	Log Fitness replicate 1	Log Fitness replicate 2
MIC_{mma} replicate 1	1.0000	0.9943	0.9972	0.9947	0.9988	0.9933	0.9682	0.9663
MIC_{mma} replicate 2	0.9943	1.0000	0.9938	0.9985	0.9947	0.9920	0.9776	0.9752
MIC_{75} replicate 1	0.9972	0.9938	1.0000	0.9964	0.9958	0.9892	0.9641	0.9624
MIC_{75} replicate 2	0.9947	0.9985	0.9964	1.0000	0.9942	0.9901	0.9731	0.9710
$MIC_{combined}$ replicate 1	0.9988	0.9947	0.9958	0.9942	1.0000	0.9945	0.9713	0.9689
$MIC_{combined}$ replicate 2	0.9933	0.9920	0.9892	0.9901	0.9945	1.0000	0.9803	0.9776
Log Fitness replicate 1	0.9682	0.9776	0.9641	0.9731	0.9713	0.9803	1.0000	0.9982
Log Fitness replicate 2	0.9663	0.9752	0.9624	0.9710	0.9689	0.9776	0.9982	1.0000

Supplementary Table 8: Correlations between predicted values derived from the stability model and the observed values of fitness/MIC for singles mutants, double mutants and epistasis across the various metrics and replicates. Pearson's r and Spearman's ρ are reported.

	Single Mutants (Pearson)	Single Mutants (Spearman)	Double Mutants (Pearson)	Double Mutants (Spearman)	Epistasis (Pearson)	Epistasis (Spearman)
MIC_{mma} replicate 1	0.9957	0.8917	0.9442	0.9327	0.7542	0.7778
MIC_{mma} replicate 2	0.9969	0.9491	0.9453	0.9349	0.7113	0.7601
MIC_{75} replicate 1	0.9962	0.8964	0.9356	0.9234	0.7506	0.7813
MIC_{75} replicate 2	0.9974	0.9453	0.9345	0.9237	0.7142	0.7716
$MIC_{combined}$ replicate 1	0.9962	0.8986	0.9387	0.9265	0.7349	0.7682
$MIC_{combined}$ replicate 2	0.9921	0.9526	0.9384	0.9340	0.6601	0.7417
Log Fitness replicate 1	0.9998	0.9824	0.9374	0.9312	0.7617	0.8156
Log Fitness replicate 2	0.9998	0.9725	0.9341	0.9273	0.7600	0.8136

Supplementary Table 9: Correlations between predicted values derived from the sum of single mutants effects and the observed values of fitness/MIC for double mutants across the various metrics and replicates. Pearson's r and Spearman's ρ are reported

A	Effects Sum (Pearson)	Effects Sum (Spearman)
MIC_{mma} replicate 1	0.8430	0.8616
MIC_{mma} replicate 2	0.8768	0.8876
MIC_{75} replicate 1	0.8367	0.8596
MIC_{75} replicate 2	0.8647	0.8786
$MIC_{combined}$ replicate 1	0.8416	0.8628
$MIC_{combined}$ replicate 2	0.8736	0.8857
Log Fitness replicate 1	0.8145	0.8725
Log Fitness replicate 2	0.8109	0.8691

Supplementary Table 10: Correlations between predicted values derived from the stability model and the observed values of fitness/MIC for double mutants for all residues pairs, the ones with distance d greater than 6 Å or lesser than 6Å. Pearson's r and Spearman's ρ are reported across the various metrics and replicates

	All Double mutants (Pearson)	All Double mutants (Spearman)	Double mutants with d<6Å (Pearson)	Double mutants with d<6Å (Spearman)	Double mutants with d>6Å (Pearson)	Double mutants with d>6Å (Spearman)
MIC_{mma} replicate 1	0.9442	0.9327	0.9332	0.9227	0.9537	0.9397
MIC_{mma} replicate 2	0.9453	0.9349	0.9333	0.9220	0.9553	0.9446
MIC_{75} replicate 1	0.9356	0.9234	0.9237	0.9125	0.9459	0.9312
MIC_{75} replicate 2	0.9345	0.9237	0.9216	0.9106	0.9451	0.9335
$MIC_{combined}$ replicate 1	0.9387	0.9265	0.9277	0.9156	0.9482	0.9342
$MIC_{combined}$ replicate 2	0.9384	0.9340	0.9263	0.9208	0.9485	0.9440
Log Fitness replicate 1	0.9374	0.9312	0.9092	0.8950	0.9562	0.9494
Log Fitness replicate 2	0.9341	0.9273	0.9057	0.8921	0.9527	0.9438

Supplementary Table 11: Correlations between predicted values of epistasis derived from the stability model and the observed values across the various metrics and replicates for all residues pairs, the ones with distance d greater than 6 Å or lesser than 6Å . Pearson's r and Spearman's ρ are reported across the various metrics and replicates.

	Epistasis All (Pearson)	Epistasis All (Spearman)	Epistasis with d<6Å (Pearson)	Epistasis with d<6Å (Spearman)	Epistasis with d>6Å (Pearson)	Epistasis with d>6Å (Spearman)
MIC_{mma} replicate 1	0.7542	0.7778	0.7316	0.7579	0.7802	0.8006
MIC_{mma} replicate 2	0.7113	0.7601	0.6916	0.7423	0.7349	0.7812
MIC_{75} replicate 1	0.7506	0.7813	0.7297	0.7583	0.7750	0.8071
MIC_{75} replicate 2	0.7142	0.7716	0.6989	0.7497	0.7326	0.7960
$MIC_{combined}$ replicate 1	0.7349	0.7682	0.7157	0.7485	0.7569	0.7908
$MIC_{combined}$ replicate 2	0.6601	0.7417	0.6397	0.7232	0.6839	0.7626
Log Fitness replicate 1	0.7617	0.8156	0.7315	0.7663	0.7975	0.8616
Log Fitness replicate 2	0.7600	0.8136	0.7324	0.7614	0.7913	0.8640

Supplementary Table 12: Estimation of the error in the stability model with a maximum likelihood model across the various metrics and replicates. The error is measured as a standard deviation (σ) in a model including all residue pairs and a single σ or a model with two error rates that are reported here, one for the pairs of sites with distance d greater than 6 Å and one for the sites with distance lesser than 6Å. The two error rates model is supported in all cases with a log likelihood improvement greater than 30 across conditions. The last column represents the ratio of estimated error between close and distant sites.

	Estimated sigma all sites	Estimated sigma d<6Å $\sigma(\mathrm{d} < 6\text{Å})$	Estimated sigma d>6Å $\sigma(\mathrm{d} > 6\text{Å})$	Ratio sigma $\frac{\sigma(\mathrm{d}<6\text{Å})}{\sigma(\mathrm{d}>6\text{Å})}$
MIC_{mma} replicate 1	0.8908	0.9507	0.8290	1.1467
MIC_{mma} replicate 2	0.8686	0.9277	0.8081	1.1480
MIC_{75} replicate 1	1.0639	1.1322	0.9942	1.1389
MIC_{75} replicate 2	1.0518	1.1158	0.9867	1.1308
$MIC_{combined}$ replicate 1	0.5276	0.5578	0.4971	1.1221
$MIC_{combined}$ replicate 2	0.5329	0.5622	0.5032	1.1173
Log Fitness replicate 1	0.0912	0.1055	0.0776	1.3593
Log Fitness replicate 2	0.0941	0.1079	0.0812	1.3295

Supplementary Table 13: Correlations between observed fitness/MIC and the ones inferred from distant homologues multiple sequence alignment through the Potts energy model (Potts) model that takes into account interaction between sites or the independent model (IND) that does not. Values are reported across metrics for single mutants as well as for double mutants. Pearson's r and Spearman's ρ are reported across the various metrics and replicates.

	Potts Single Mutants (Pearson)	Potts Single Mutants (Spearman)	Potts Double Mutants (Pearson)	Potts Double Mutants (Spearman)	IND Single Mutants (Pearson)	IND Single Mutants (Spearman)	IND Double Mutants (Pearson)	IND Double Mutants (Spearman)
MIC_{mma} replicate 1	0.6517	0.7447	0.7419	0.7646	0.5819	0.6086	0.6541	0.6622
MIC_{mma} replicate 2	0.6797	0.8098	0.7531	0.7749	0.6071	0.6456	0.6635	0.6746
MIC_{75} replicate 1	0.6475	0.7503	0.7338	0.7591	0.5788	0.6037	0.6474	0.6572
MIC_{75} replicate 2	0.6730	0.8073	0.7441	0.7683	0.6030	0.6434	0.6563	0.6684
$MIC_{combined}$ replicate 1	0.6496	0.7477	0.7383	0.7623	0.5804	0.6074	0.6511	0.6602
$MIC_{combined}$ replicate 2	0.6765	0.8088	0.7491	0.7720	0.6051	0.6437	0.6603	0.6719
Log Fitness replicate 1	0.7683	0.8491	0.7126	0.7304	0.6753	0.7344	0.6090	0.6185
Log Fitness replicate 2	0.7689	0.8462	0.7127	0.7303	0.6765	0.7282	0.6096	0.6196

Supplementary Table 14: Correlations between observed epistasis and the ones inferred from distant homologues multiple sequence alignment through the Potts energy models (Potts score) that takes into account interaction between sites. Values are reported across metrics for single mutants as well as for double mutants. Pearson's r and Spearman's ρ are reported.

	Epistasis (Pearson)	Epistasis (Spearman)
MIC_{mma} replicate 1	-0.0060	-0.0166
MIC_{mma} replicate 2	0.0107	-0.0045
MIC_{75} replicate 1	-0.0029	-0.0165
MIC_{75} replicate 2	0.0118	-0.0081
$MIC_{combined}$ replicate 1	-0.0037	-0.0157
$MIC_{combined}$ replicate 2	0.0128	-0.0053
Log Fitness replicate 1	0.0361	0.0201
Log Fitness replicate 2	0.0368	0.0165

Supplementary Table 15: Correlations between inferred singles mutants ΔΔG and the fitness inferred from distant homologues multiple sequence alignment through the Potts energy model (Potts) that takes into account interaction between sites or the independent model (IND) that does not. Values are reported across metrics for single mutants. Pearson's r and Spearman's ρ are reported.

	Potts score to ΔΔG (Pearson)	Potts score to ΔΔG (Spearman)	IND score to ΔΔG (Pearson)	IND score to ΔΔG (Spearman)
MIC_{mma} replicate 1	-0.8286	-0.8499	-0.7189	-0.7449
MIC_{mma} replicate 2	-0.8300	-0.8507	-0.7165	-0.7409
MIC_{75} replicate 1	-0.8276	-0.8471	-0.7213	-0.7466
MIC_{75} replicate 2	-0.8308	-0.8466	-0.7229	-0.7419
$MIC_{combined}$ replicate 1	-0.8230	-0.8508	-0.7090	-0.7447
$MIC_{combined}$ replicate 2	-0.7712	-0.8527	-0.6830	-0.7547
Log Fitness replicate 1	-0.8057	-0.7994	-0.6104	-0.6077
Log Fitness replicate 2	-0.8057	-0.8005	-0.6191	-0.6121

Supplementary Table 16: Models parameters to rescale Potts model energy into free energies. Mutants whose mutation have been seen in the MSA that were non-lethal were used. The transformations of Potts energies into $\Delta\Delta G^{Potts}$ and an associated ΔG_0^{Potts} was done in three ways. First, Model 1, the scaling parameter γ (Equation 8) was computed from the regression between Potts energies and the inferred free energies ΔΔG and we used the inferred wild type free energy: $\Delta\Delta G^{Potts} = \Delta G_0$. Second, Model 2, using the fitness values of the single mutants, ΔG_0^{Potts} and the scaling parameter γ were optimized to maximize the log likelihood of the observed fitness value and the one derived from the scaling of the Potts energies plugged in the two-states model: $\sum_{single\ mutants} \frac{\left(f_i^{observed} - f_i^{predicted}\right)^2}{2\left(sd_i^{observed}\right)^2}$ with $f_i^{observed}$ the observed Log fitness (or MIC), $f_i^{predicted} = log\left(1 + e^{\frac{\Delta G_0^{Potts}}{RT}}\right) - log\left(1 + e^{\frac{\Delta G_0^{Potts} + \gamma\ Potts(\mathrm{i})}{RT}}\right)$ is the predicted fitness from the rescaled Potts energies and $sd_i^{observed}$ the standard deviation of the fitness measurement of mutant i. Third, Model 3, the same maximum likelihood method was used but this time on all mutants and not just the single ones.

	Model 1: ΔG_0 from stability model	Model 2: Estimated ΔG_0^{Potts} from Likelihood model using single mutants	Model 3: Estimated ΔG_0^{Potts} from Likelihood model using all mutants	Model 1: Fitted scaling parameter γ of Potts energies to infered ΔΔG of single mutants	Model 2: Estimated scaling parameter γ from Likelihood model using single mutants	Model 3: Estimated scaling parameter γ from Likelihood model using all mutants
MIC_{mma} replicate 1	-3.45	-4.2578	-0.7880	-0.5999	-0.8058	-0.3118
MIC_{mma} replicate 2	-2.65	-3.9323	-0.7086	-0.5164	-0.7703	-0.3111
MIC_{75} replicate 1	-4.00	-4.7146	-0.0896	-0.6891	-0.9066	-0.2985
MIC_{75} replicate 2	-3.12	-4.3229	0.0350	-0.5952	-0.8579	-0.2962
$MIC_{combined}$ replicate 1	-3.20	-4.1052	-1.2987	-0.4650	-0.6226	-0.2373
$MIC_{combined}$ replicate 2	-2.35	-3.6171	-1.2155	-0.3820	-0.5654	-0.2355
Log Fitness replicate 1	-4.50	-3.2994	-2.3019	-0.5947	-0.3768	-0.1731
Log Fitness replicate 2	-4.92	-3.3346	-2.2898	-0.6642	-0.3816	-0.1723

Supplementary Table 17: Correlation between the measured fitness/MIC and the estimates resulting from the rescaling of the Potts energy (see Supplementary Table 16 for details). Here, only non-lethal mutants seen in the MSA are retained. As the rescaling does not affect rank, Spearman's (correlation are minimally affected (a bit through the thresholding), therefore only Pearson's r are reported.

	Model 1 (Pearson)	Model 2 (Pearson)	Model 3 (Pearson)
MIC_{mma} replicate 1	0.7394	0.7447	0.7501
MIC_{mma} replicate 2	0.7520	0.7527	0.7595
MIC_{75} replicate 1	0.7316	0.7387	0.7407
MIC_{75} replicate 2	0.7437	0.7461	0.7498
$MIC_{combined}$ replicate 1	0.7200	0.7217	0.7402
$MIC_{combined}$ replicate 2	0.7382	0.7304	0.7486
Log Fitness replicate 1	0.4448	0.4710	0.5426
Log Fitness replicate 2	0.4425	0.4729	0.5471

Supplementary Table 18: Correlation between observed epistasis and the one resulting from the rescaling of the Potts energy (see Supplementary Table 16 for details). Only non-lethal mutants seen in the MSA are retained. Pearson's r and Spearman' ρ are reported.

	Model 1 Epistasis (Pearson)	Model 1 Epistasis (Spearman)	Model 2 Epistasis (Pearson)	Model 2 Epistasis (Spearman)	Model 3 Epistasis (Pearson)	Model 3 Epistasis (Spearman)
MIC_{mma} replicate 1	0.5008	0.5306	0.4119	0.4453	0.5315	0.5651
MIC_{mma} replicate 2	0.5155	0.5499	0.4305	0.4695	0.5292	0.5678
MIC_{75} replicate 1	0.4900	0.5282	0.3928	0.4363	0.4574	0.4989
MIC_{75} replicate 2	0.5046	0.5519	0.4109	0.4646	0.4400	0.4871
$MIC_{combined}$ replicate 1	0.4613	0.5105	0.3934	0.4479	0.5342	0.5752
$MIC_{combined}$ replicate 2	0.4807	0.5306	0.4149	0.4742	0.5254	0.5765
Log Fitness replicate 1	0.3639	0.4450	0.3815	0.4716	0.4569	0.5260
Log Fitness replicate 2	0.3461	0.4346	0.3814	0.4732	0.4628	0.5284

Supplementary Table 19: Primers

**Plasmid construction**	
TEM-siteNcoI-avant-Halpha	agaaaagcatcttacggatgCcatgGcagtaagagaattatgcagt
pSkunk-ins-NotI-aval-CmR	cgatcaacgtctcattttcgccaaaagGCggccGCgggcttcccggtatcaacagggacacca
pSkunk-ins-XhoI-avalTEM	tgggctacgtcttgctggcgttcggggtcgaatCTCGAGttgctttcgaatttctgccattcat
**Mutagenesis**	
TEM-H4-1-2	atcttacggatggcatgacaNNSNNSgaattatgcagtgctgccataaccatgagtgataacactgcggccaa
TEM-H4-1-3	atcttacggatggcatgacaNNSagaNNSttatgcagtgctgccataaccatgagtgataacactgcggccaa
TEM-H4-1-4	atcttacggatggcatgacaNNSagagaaNNStgcagtgctgccataaccatgagtgataacactgcggccaa
TEM-H4-1-5	atcttacggatggcatgacaNNSagagaattaNNSagtgctgccataaccatgagtgataacactgcggccaa
TEM-H4-1-6	atcttacggatggcatgacaNNSagagaattatgcNNSgctgccataaccatgagtgataacactgcggccaa
TEM-H4-1-7	atcttacggatggcatgacaNNSagagaattatgcagtNNSgccataaccatgagtgataacactgcggccaa
TEM-H4-1-8	atcttacggatggcatgacaNNSagagaattatgcagtgctNNSataaccatgagtgataacactgcggccaa
TEM-H4-1-9	atcttacggatggcatgacaNNSagagaattatgcagtgctgccNNSaccatgagtgataacactgcggccaa
TEM-H4-1-10	atcttacggatggcatgacaNNSagagaattatgcagtgctgccataNNSatgagtgataacactgcggccaa

TEM-H4-1-11	atcttacggatggcatgacaNNSagagaattatgcagtgctgccataaccNNSagtgataacactgcggccaa
TEM-H4-2-3	atcttacggatggcatgacagtaNNSNNSttatgcagtgctgccataaccatgagtgataacactgcggccaa
TEM-H4-2-4	atcttacggatggcatgacagtaNNSgaaNNStgcagtgctgccataaccatgagtgataacactgcggccaa
TEM-H4-2-5	atcttacggatggcatgacagtaNNSgaattaNNSagtgctgccataaccatgagtgataacactgcggccaa
TEM-H4-2-6	atcttacggatggcatgacagtaNNSgaattatgcNNSgctgccataaccatgagtgataacactgcggccaa
TEM-H4-2-7	atcttacggatggcatgacagtaNNSgaattatgcagtNNSgccataaccatgagtgataacactgcggccaa
TEM-H4-2-8	atcttacggatggcatgacagtaNNSgaattatgcagtgctNNSataaccatgagtgataacactgcggccaa
TEM-H4-2-9	atcttacggatggcatgacagtaNNSgaattatgcagtgctgccNNSaccatgagtgataacactgcggccaa
TEM-H4-2-10	atcttacggatggcatgacagtaNNSgaattatgcagtgctgccataNNSatgagtgataacactgcggccaa
TEM-H4-2-11	atcttacggatggcatgacagtaNNSgaattatgcagtgctgccataaccNNSagtgataacactgcggccaa
TEM-H4-3-4	atcttacggatggcatgacagtaagaNNSNNStgcagtgctgccataaccatgagtgataacactgcggccaa
TEM-H4-3-5	atcttacggatggcatgacagtaagaNNSttaNNSagtgctgccataaccatgagtgataacactgcggccaa
TEM-H4-3-6	atcttacggatggcatgacagtaagaNNSttatgcNNSgctgccataaccatgagtgataacactgcggccaa
TEM-H4-3-7	atcttacggatggcatgacagtaagaNNSttatgcagtNNSgccataaccatgagtgataacactgcggccaa
TEM-H4-3-8	atcttacggatggcatgacagtaagaNNSttatgcagtgctNNSataaccatgagtgataacactgcggccaa
TEM-H4-3-9	atcttacggatggcatgacagtaagaNNSttatgcagtgctgccNNSaccatgagtgataacactgcggccaa
TEM-H4-3-10	atcttacggatggcatgacagtaagaNNSttatgcagtgctgccataNNSatgagtgataacactgcggccaa
TEM-H4-3-11	atcttacggatggcatgacagtaagaNNSttatgcagtgctgccataaccNNSagtgataacactgcggccaa
TEM-H4-4-5	atcttacggatggcatgacagtaagagaaNNSNNSagtgctgccataaccatgagtgataacactgcggccaa
TEM-H4-4-6	atcttacggatggcatgacagtaagagaaNNStgcNNSgctgccataaccatgagtgataacactgcggccaa
TEM-H4-4-7	atcttacggatggcatgacagtaagagaaNNStgcagtNNSgccataaccatgagtgataacactgcggccaa
TEM-H4-4-8	atcttacggatggcatgacagtaagagaaNNStgcagtgctNNSataaccatgagtgataacactgcggccaa
TEM-H4-4-9	atcttacggatggcatgacagtaagagaaNNStgcagtgctgccNNSaccatgagtgataacactgcggccaa
TEM-H4-4-10	atcttacggatggcatgacagtaagagaaNNStgcagtgctgccataNNSatgagtgataacactgcggccaa
TEM-H4-4-11	atcttacggatggcatgacagtaagagaaNNStgcagtgctgccataaccNNSagtgataacactgcggccaa
TEM-H4-5-6	atcttacggatggcatgacagtaagagaattaNNSNNSgctgccataaccatgagtgataacactgcggccaa
TEM-H4-5-7	atcttacggatggcatgacagtaagagaattaNNSagtNNSgccataaccatgagtgataacactgcggccaa
TEM-H4-5-8	atcttacggatggcatgacagtaagagaattaNNSagtgctNNSataaccatgagtgataacactgcggccaa
TEM-H4-5-9	atcttacggatggcatgacagtaagagaattaNNSagtgctgccNNSaccatgagtgataacactgcggccaa
TEM-H4-5-10	atcttacggatggcatgacagtaagagaattaNNSagtgctgccataNNSatgagtgataacactgcggccaa
TEM-H4-5-11	atcttacggatggcatgacagtaagagaattaNNSagtgctgccataaccNNSagtgataacactgcggccaa
TEM-H4-6-7	atcttacggatggcatgacagtaagagaattatgcNNSNNSgccataaccatgagtgataacactgcggccaa
TEM-H4-6-8	atcttacggatggcatgacagtaagagaattatgcNNSgctNNSataaccatgagtgataacactgcggccaa
TEM-H4-6-9	atcttacggatggcatgacagtaagagaattatgcNNSgctgccNNSaccatgagtgataacactgcggccaa
TEM-H4-6-10	atcttacggatggcatgacagtaagagaattatgcNNSgctgccataNNSatgagtgataacactgcggccaa
TEM-H4-6-11	atcttacggatggcatgacagtaagagaattatgcNNSgctgccataaccNNSagtgataacactgcggccaa
TEM-H4-7-8	atcttacggatggcatgacagtaagagaattatgcagtNNSNNSataaccatgagtgataacactgcggccaa
TEM-H4-7-9	atcttacggatggcatgacagtaagagaattatgcagtNNSgccNNSaccatgagtgataacactgcggccaa
TEM-H4-7-10	atcttacggatggcatgacagtaagagaattatgcagtNNSgccataNNSatgagtgataacactgcggccaa
TEM-H4-7-11	atcttacggatggcatgacagtaagagaattatgcagtNNSgccataaccNNSagtgataacactgcggccaa
TEM-H4-8-9	atcttacggatggcatgacagtaagagaattatgcagtgctNNSNNSaccatgagtgataacactgcggccaa
TEM-H4-8-10	atcttacggatggcatgacagtaagagaattatgcagtgctNNSataNNSatgagtgataacactgcggccaa
TEM-H4-8-11	atcttacggatggcatgacagtaagagaattatgcagtgctNNSataaccNNSagtgataacactgcggccaa
TEM-H4-9-10	atcttacggatggcatgacagtaagagaattatgcagtgctgccNNSNNSatgagtgataacactgcggccaa
TEM-H4-9-11	atcttacggatggcatgacagtaagagaattatgcagtgctgccNNSaccNNSagtgataacactgcggccaa
TEM-H4-10-11	atcttacggatggcatgacagtaagagaattatgcagtgctgccataNNSNNSagtgataacactgcggccaa

P320	gcagaaattcgaaagcaaattcgac

Barcoding part

PKD3-Cm-XhoI-F	ttgctggcgttcggggtcgatctcgagttgctttcgaatNNNNNNNNNNAC NNNNNNNNNNTTacgccccgccctgccactcatcgcagtact
PKD3-cm-NotI-R	cctggtgtccctgttgataccgggaagcccgcggccgcaattagccatggtccatatgaatatcctccttag

Sequencing part

heliceA-BC-NGS-F	tcgtcggcagcgtcagatgtgtataagagacagNNNNNNaagcatcttacggatggcatga
NGS-BC-Gibson-R	gtctcgtgggctcggagatgtgtataagagacagNNNNNNattacaacagtactgcgatgagtggcag
NGSavt-BC-bis2-F	tcgtcggcagcgtcagatgtgtataagagacagNNNNNNaccggccgacgcgctgggctacgtcttgct

Supplementary Table 20: Number of colonies at different concentration of antibiotics

	Antibiotic Lot A		Antibiotic Lot B		
Concentration (mg/l)	Library 1	Library 2	Library 3	Library 4	Median
0	1.15E+08	1.05E+08	1.00E+08	1.13E+08	1.09E+08
0.125	3.45E+07	3.68E+07	4.60E+07	3.03E+07	3.57E+07
0.25	3.28E+07	3.53E+07	2.40E+07	2.50E+07	2.89E+07
0.5	2.70E+07	3.10E+07	2.65E+07	2.35E+07	2.68E+07
1	1.83E+07	2.25E+07	2.35E+07	2.20E+07	2.23E+07
2	1.70E+07	2.08E+07	2.10E+07	1.85E+07	1.97E+07
4	1.48E+07	1.43E+07	1.05E+07	1.40E+07	1.42E+07
8	1.43E+07	1.38E+07	1.20E+07	1.30E+07	1.34E+07
16	6.73E+06	5.28E+06	5.50E+06	1.00E+07	6.12E+06
32	4.20E+05	3.40E+05	7.50E+05	7.50E+05	5.85E+05
64	0.00E+00	0.00E+00	0.00E+00	0.00E+00	0.00E+00

Supplementary Table 21: Comparison between T_k and T_k^{WT} as explained in Supplementary Note 1.

k	0	1	2	4	6
T_k	0	4	9	19	29
T_k^{WT}	0	6.6	11.9	22.0	32.1

Supplementary figures

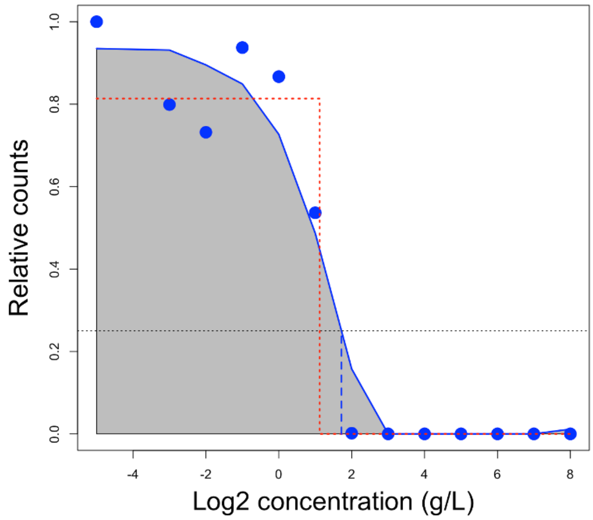

Supplementary Figure S1. Illustration of MIC metrics. The read counts multiplied by the plate counts and normalized to the no antibiotic control are plotted along plating concentrations. Blue dots represent real data, the blue line the smoothed fit. The gray area corresponds to a measure of MIC, MIC_{Area}, the intercept between the blue curve and the horizontal dotted line at 0.25 corresponds to MIC_{EC75}. The red dotted line represents a moment matching approach attempt to describe the blue points through a step function, the transition concentration corresponding to MIC_{MMA}. MIC_{Area} being very noisy it was not analysed further.

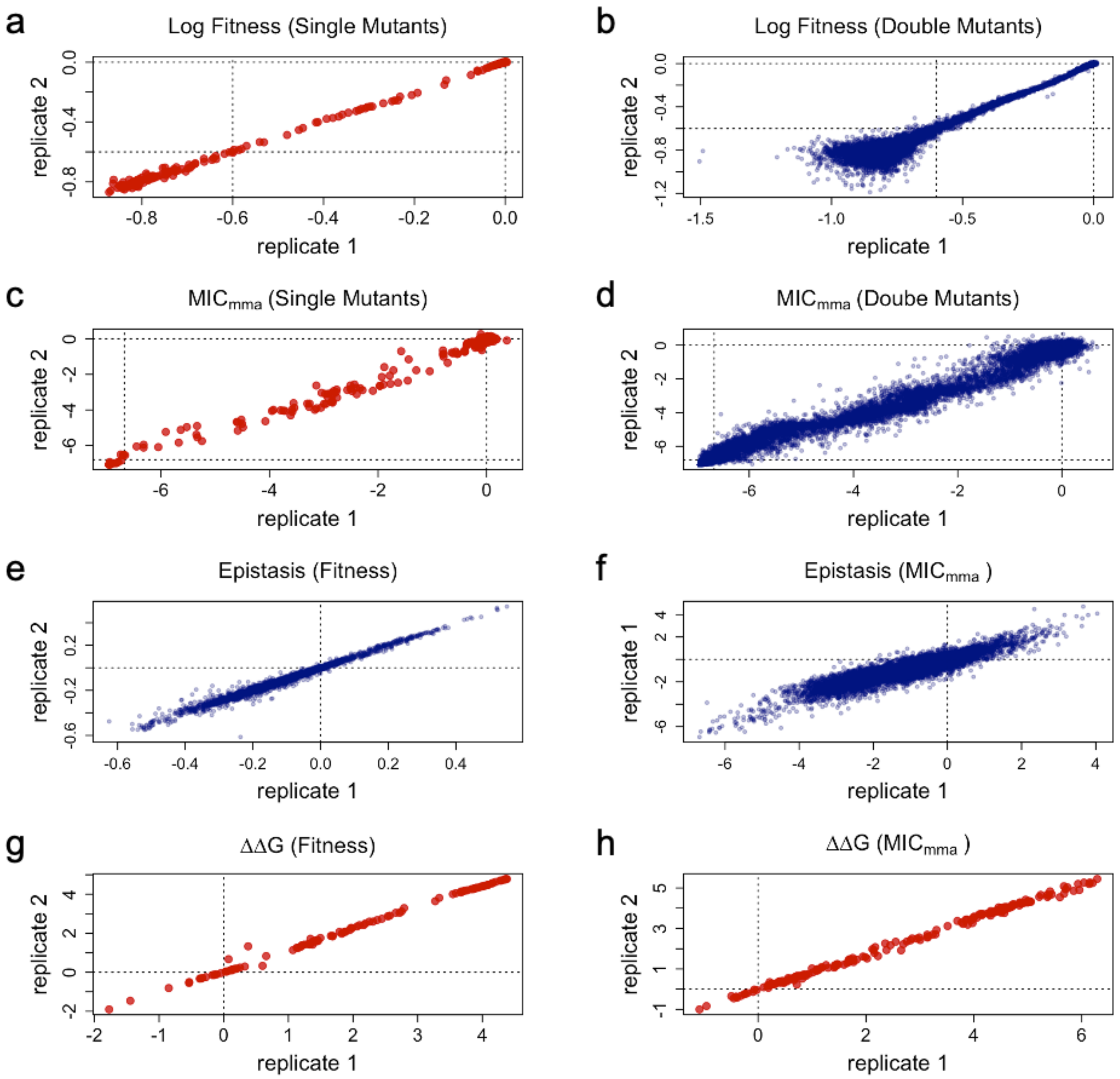

Supplementary Figure S2: Comparison between two biological semi-replicates. (a) Single mutants' log Fitness. (b) Double mutants' log Fitness, (c) Single mutants' MIC_{mma}, (d) Double mutants' MIC_{mma}, (e) Epistasis computed on fitness, (f) Epistasis computed on MIC_{mma},(g) $\Delta\Delta G$ computed on fitness, (h) $\Delta\Delta G$ computed on MIC_{mma}. Source data are provided as a Source Data file.

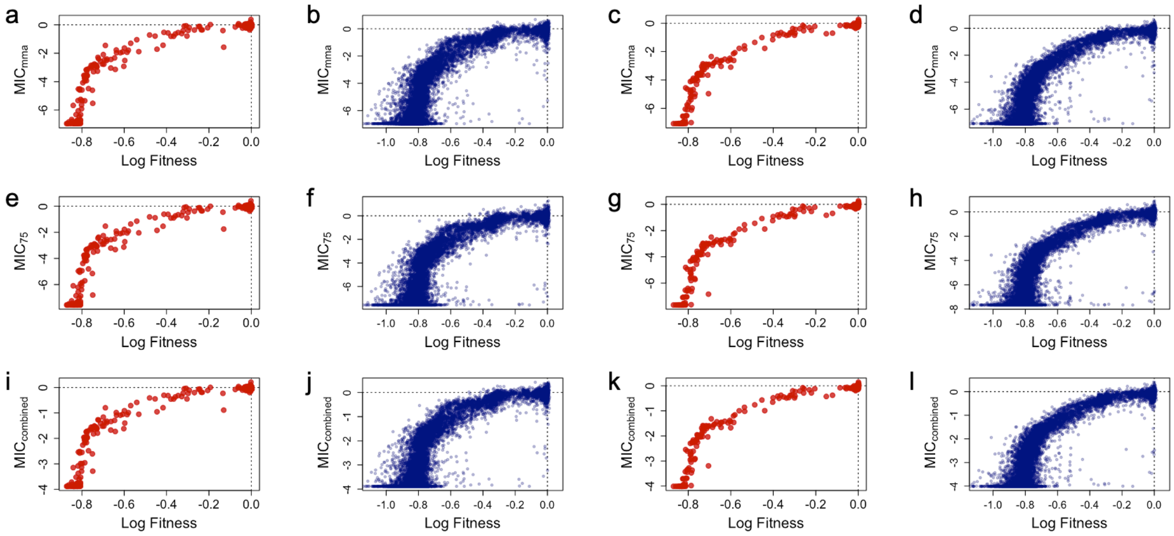

Supplementary Figure S3: Comparison between log Fitness and the different MIC scores for single (a, c, e, g, i, k) and double mutants (b, d, f, h , l), for the first replicate (a, b, e, f, i, j) and second replicate (c, d, g, h, k, l). Source data are provided as a Source Data file.

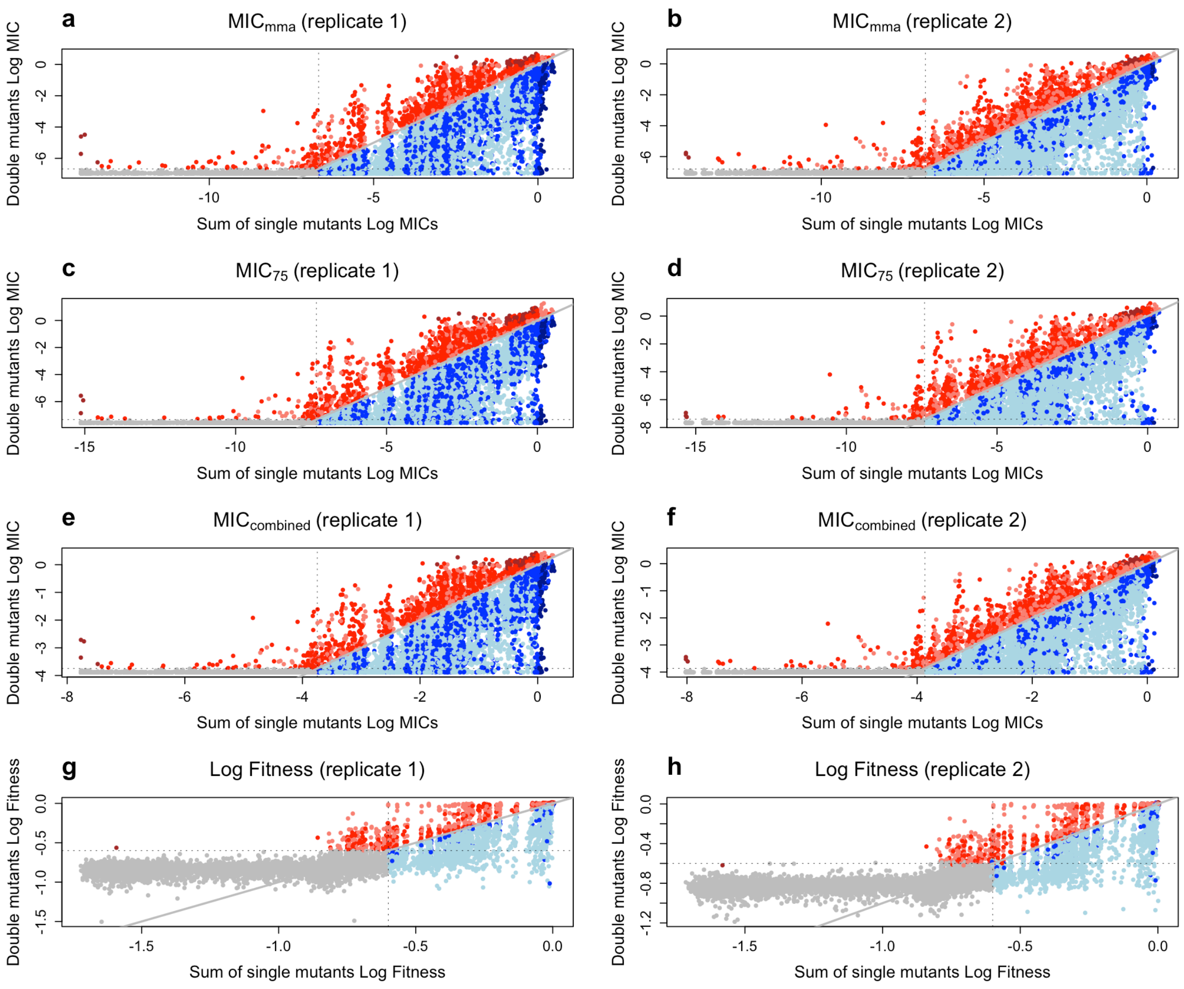

Supplementary Figure S4 : Effect of double mutants compared to the effect of the sum of the single mutants for the different measures of log MIC of Log Fitness and both replicates (A to G). Dotted lines represent the value below which mutants are not different from non-functional mutants. Points are colored according to the double mutant's epistasis type as presented in figure 1A. Very dark blue represents reciprocal negative sign epistasis; blue: negative sign epistasis; light blue: negative epistasis; grey: mutants for which epistasis cannot be measured; light red: positive epistasis, red: positive sign epistasis and dark red: positive reciprocal sign epistasis. Source data are provided as a Source Data file.

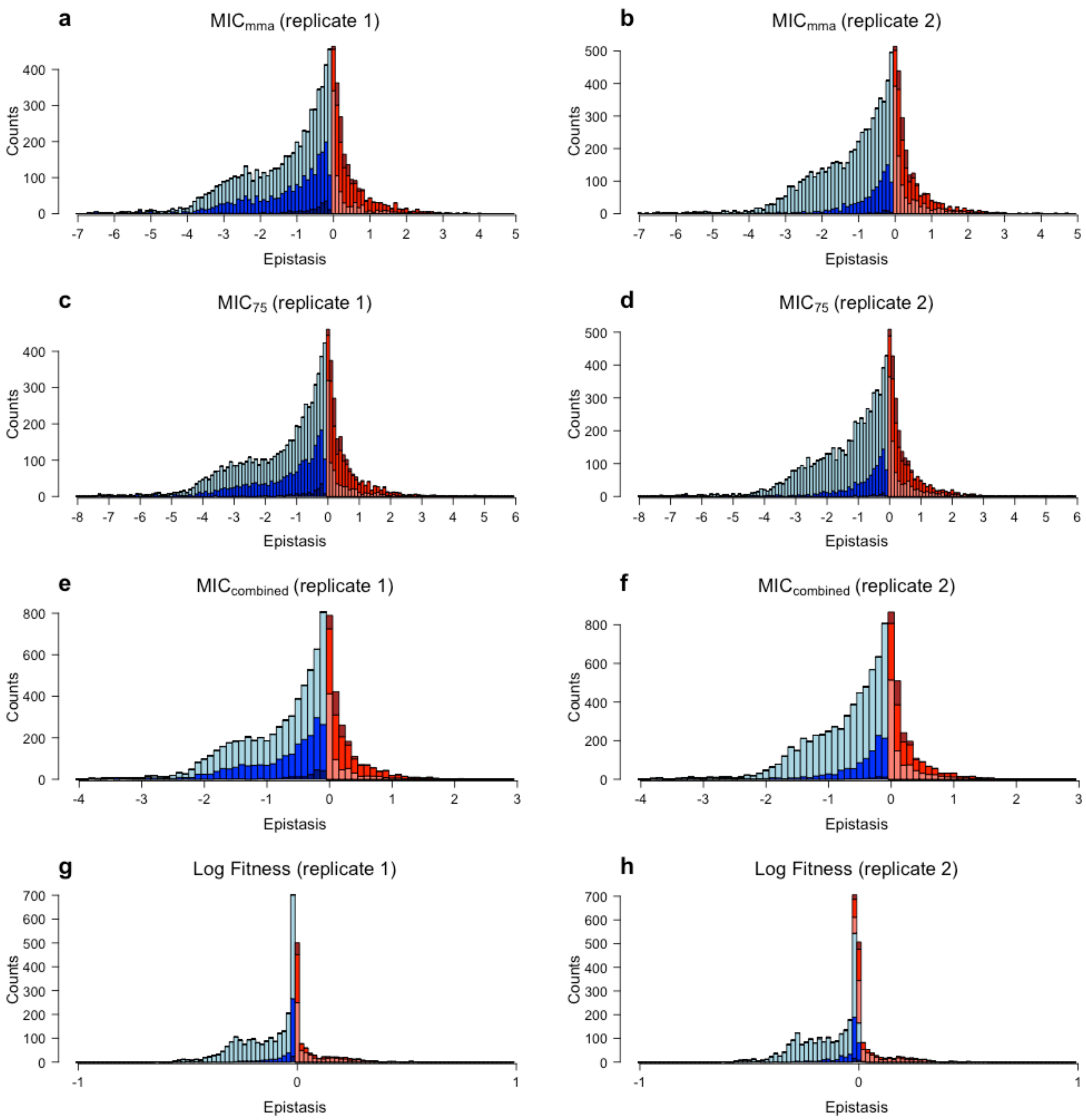

Supplementary Figure S5 : Distribution of epistasis derived from log MICs or Log Fitness estimates for both replicates (A to G). Epistasis is plotted for mutants for which it could be quantitatively estimated, i.e, neither singles nor double mutants's values were below the functional threshold. The colors represent the type of epistasis present in each bin of the distribution. Very dark blue represents reciprocal negative sign epistasis; blue: negative sign epistasis; light blue: negative epistasis; light red: positive epistasis, red: positive sign epistasis and dark red: positive reciprocal sign epistasis. Source data are provided as a Source Data file.

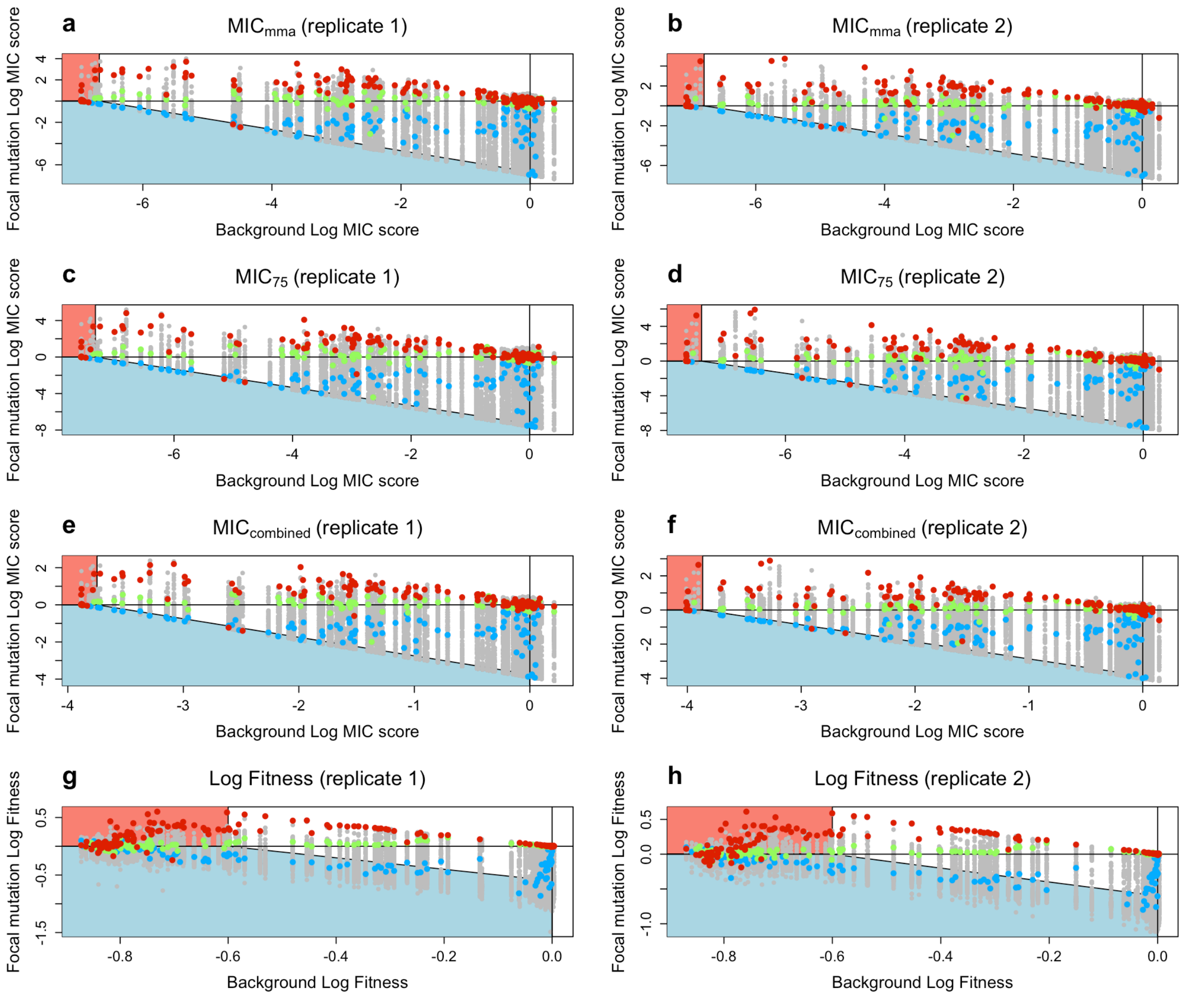

Supplementary Figure S6 : Effect of mutations according to the log MIC or Log Fitness of the background for the different metrics and replicates. The log MIC or Log Fitness impact of a focal mutation is plotted against the log MIC or Log Fitness of the genotype to which it is added. Three specific focal mutations with contrasted patterns are colored: R120V in blue, R120Q in green and S124E in red. The regions in blue represents mutants with log MIC or Log Fitness below the threshold corresponding to non-functional alleles, the salmon region represent below threshold mutant backgrounds that when associated from with a stabilizing mutation have a detectable Log MIC or Log Fitness. Source data are provided as a Source Data file.

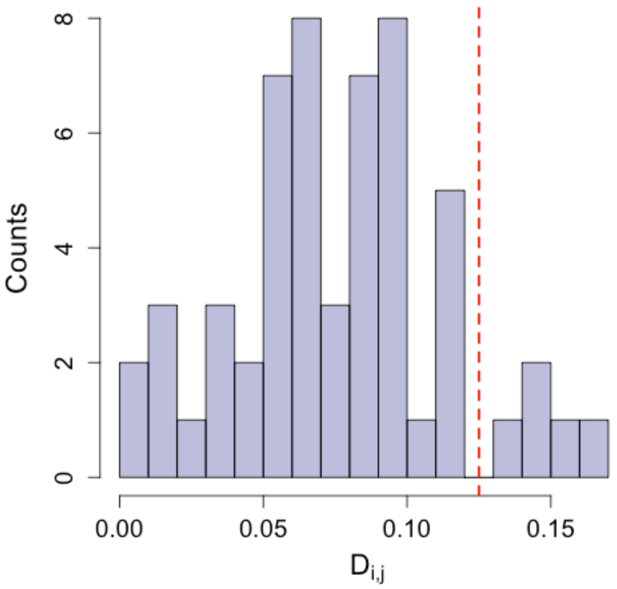

Supplementary Figure S7: Distribution of D_{ij}. Red dashed line separates the top five pairs of sites from the others. Source data are provided as a Source Data file.

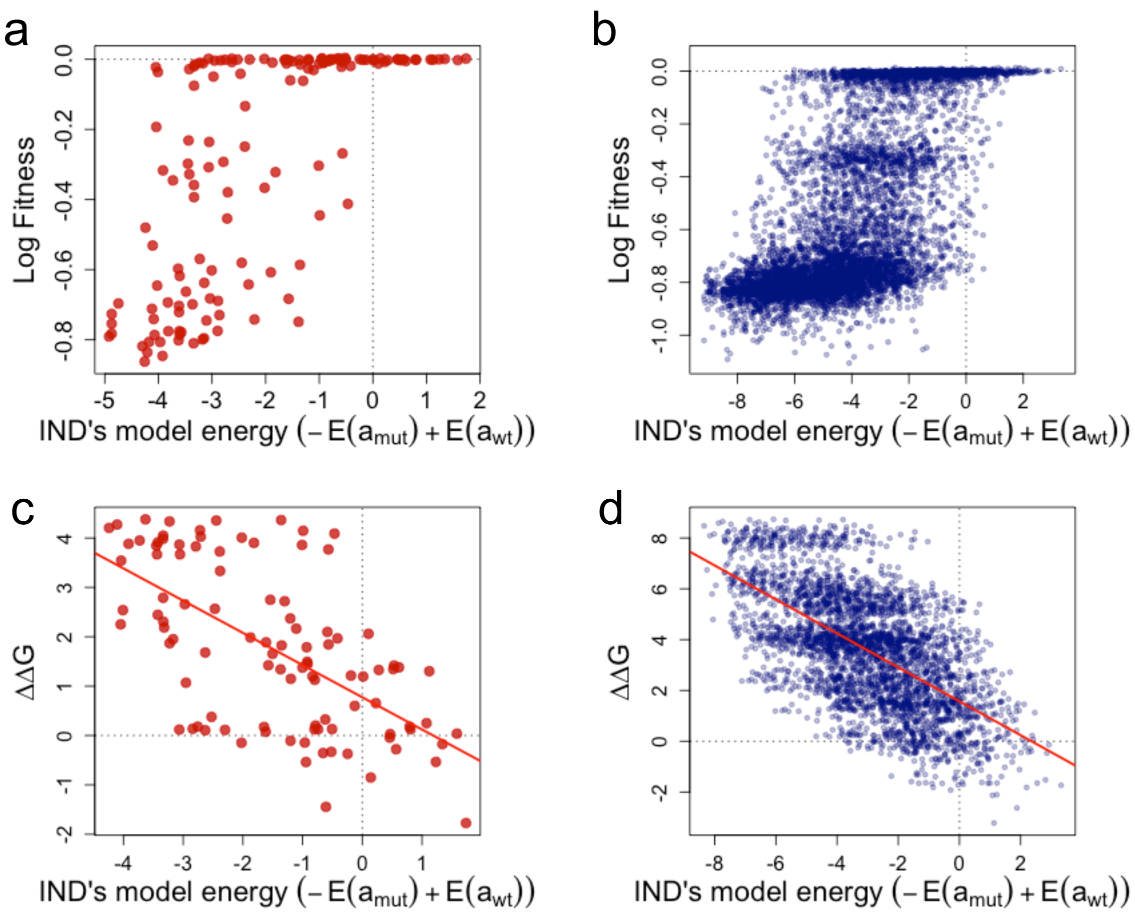

Supplementary Figure S8 : Independent model's energies versus experimental quantities. (a) Experimental log-fitness against $-E\left(a_{mut_i^a}\right) + E(a_{WT})$ for single mutants. (b) Experimental log-fitness against $-E\left(a_{mut_{ij}^{ab}}\right) + E(a_{WT})$ for double mutants. (c) $\Delta\Delta G_i^a$ against $-E\left(a_{mut_i^a}\right) + E(a_{WT})$ (d) $\Delta\Delta G_i^a + \Delta\Delta G_j^b$ against $-E\left(a_{mut_{ij}^{ab}}\right) + E(a_{WT})$. Source data are provided as a Source Data file.

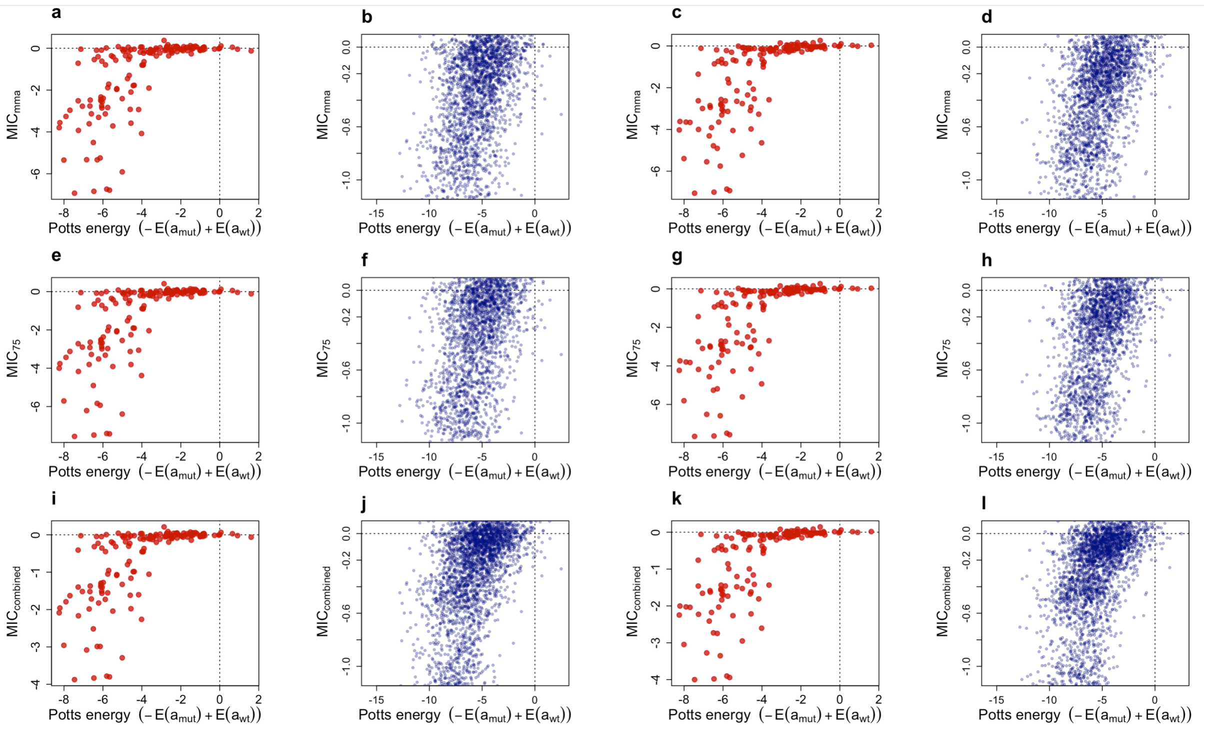

Supplementary Figure S9 : Potts model's energies versus experimental quantities. (a, c, e, g, i, k) Experimental MIC measures (a, e, i are first replicate, c, g, k the second replicate, a,c MIC_{mma}, e, g, MIC_{75}, i, k, $MIC_{combined}$), against $-E\left(a_{mut_i^a}\right) + E(a_{WT})$ for single mutants. (b) Experimental MIC measures (b, f, j are first replicate, d, h, i the second replicate, b, d MIC_{mma}, f, h MIC_{75}, j, l, $MIC_{combined}$) against $-E\left(a_{mut_{ij}^{ab}}\right) + E(a_{WT})$ for double mutants. Source data are provided as a Source Data file.

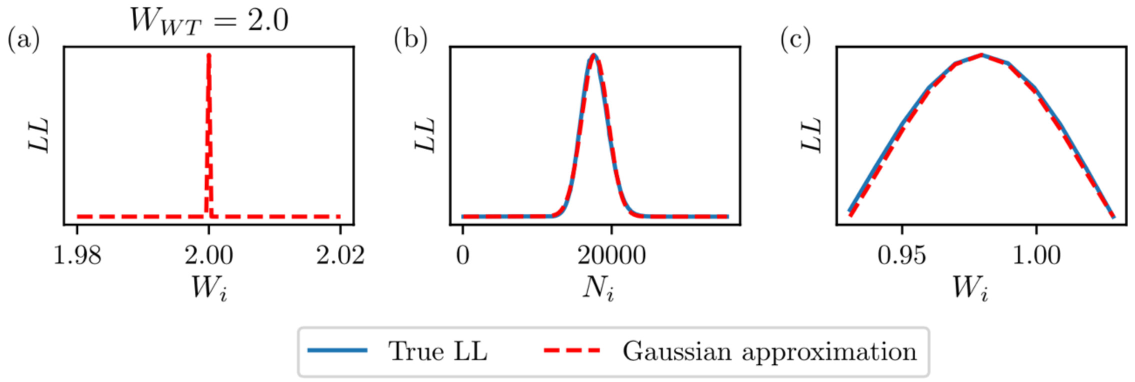

Supplementary Figure S10: Quality of the Gaussian approximation for the fitness inference. (a) Estimation of $\boldsymbol{W_{WT}}$. $\boldsymbol{W_{WT} = 2}$ in agreement with the definition of the times $\boldsymbol{T_k}$. (b) and (c) Comparison between the exact log-likelihood (LL) and the Gaussian approximation for a given mutant for the estimation of $\boldsymbol{N_i(0)}$ (b) and of $\boldsymbol{W_i}$ (c).

Supplementary Notes

Supplementary Note 1: Inference of the log-fitness

In this section, we detail the inference procedure we developed to estimate the log-fitness of each mutant.

Modeling of the DNA sequencer

To infer the fitness of each mutation i, we model the evolution of the number of plasmids over time. We denote the actual population of plasmids carrying the mutation i at time (step) T_k by $N_i(T_k)$. According to the definition of absolute fitness, $N_i(T_k)$ follows an exponential growth:

$$N_i(t+1) = W_i \times N_i(t), \tag{1}$$

We use similar notations for the wild-type (WT) sequence, with $N_{WT}(t)$ and W_{WT} for, respectively the population size and the fitness of the WT. We introduce the relative fitness with respect to the wild-type (WT),

$$w_i = \frac{W_i}{W_{WT}}. \tag{2}$$

According to the experimental protocol, due to the different dilutions after each measure, the population of plasmids carrying mutation i at time T_k can be written as

$$N_i(T_k) = d^k \; W_i^{T_k} \times N_i(0), \tag{3}$$

where $d = \frac{1}{32}$ is the dilution factor.

However, we do not have directly access to $N_i(T_k)$ but to the output of the DNA sequencer, $\widehat{N}_i(T_k)$ (notice that the different barcodes coding for the same mutation are grouped together). The DNA sequencer does not sample all the plasmids, but a fraction of them only. For simplicity, we assume that each measurement of $\widehat{N}_i(T_k)$ is a realization of the binomial distribution $\mathcal{B}(N_i(T_k)\,, p_k)$, where p_k is the sampling rate of the DNA sequencer at time T_k. We estimate this sampling rate through

$$p_k = \frac{\widehat{N}(T_k)}{N_{OD}(T_k)}\,, \tag{4}$$

where $\widehat{N}(T_k)$ is the total number of samples (WT and variants) provided by the DNA sequencer at time T_k, and $N_{OD}(T_k)$ is the total population at time T_k.

Time parameterization based on WT evolution

According to the measurement protocol, the different times steps T_k correspond to numbers of population-averaged generations, which are reported in the first row of Supplementary Table 21. Notice that the dilution factor $d = \frac{1}{32} = \frac{1}{2^5}$ corresponds to 5 population-averaged doublings, which explains why $T_{k+1} - T_k = 5$, except for $T_1 - T_0 = 4$, since, at T_0, $OD_{600} = 0.4$, and only 4 population-averaged doublings are needed to reach $OD_{600} = 0.2$ upon dilution.

The total number of plasmids theoretically evolves as

$$N(T_k) = 2^{T_k - T_{k-1}} \times N(T_{k-1}) \,. \tag{5}$$

However, as a significant fraction of mutants may die due to the antibiotic, the number of generations may be underestimated during a cycle. Therefore, we redefined the time scale by the number of generations of the WT. With this new definition of time T_k^{WT}, the absolute fitness of the wild-type, W_{WT}, is equal to 2.

We determine the relation between the times T_k^{WT} and T_k as follows. Let $\widehat{N}^{WT}(T_k)$ be the population of WT at time T_k measured by the DNA sequencer, and $f(T_k)$ be the frequency of WT after the kth cycle,

$$f(T_k) = \frac{\widehat{N}^{WT}(T_k)}{\widehat{N}(T_k)} \,. \tag{6}$$

As, according to Equation 5,

$$\widehat{N}(T_k) = 2^{T_k - T_{k-1}} \times \widehat{N}(T_{k-1}) \,, \tag{7}$$

and, similarly,

$$\widehat{N}^{WT}(T_k) = 2^{T_k^{WT} - T_{k-1}^{WT}} \times \widehat{N}(T_{k-1}) \,, \tag{8}$$

we get,

$$T_k^{WT} - T_{k-1}^{WT} = T_k - T_{k-1} + \log_2 \frac{f(T_k)}{f(T_{k-1})} \,, \tag{9}$$

and, by summing over k starting from 0,

$$T_k^{WT} \quad = \quad T_k + \log_2 \frac{f(T_k)}{f(T_0)} \; . \qquad (10)$$

Values for $t_k^{WT}$are reported in Supplementary Table 21, second row. In the following, we use T_k^{WT} as reference time scale, but we note it as T_k for the sake of simplify.

Computation of the likelihood

For a given mutation i, we want to estimate the absolute fitness W_i knowing the measurements of the population $\{\widehat{N}_i(T_k)\}_k$ at different times T_k. Based on the modeling of the sampling carried out by the DNA sequencer above, the probability of these measurements can be written as

$$P(\{\widehat{N}_i(T_k)\}_k|W_i) \propto \sum_{N_i(0)=0}^{\infty} \prod_{k=0}^{k_f} \binom{d^k {W_i}^{T_k} N_i(0)}{\widehat{N}_i(T_k)} p_k^{\widehat{N}_i(T_k)} (1-p_k)^{d^k {W_i}^{T_k} N_i(0) - \widehat{N}_i(T_k)} \; , \quad (11)$$

where k_f is the number of cycles considered. In practice, we set $k_f = 2$, as after 2 cycles of intense selection, the trajectories of log frequencies relative to wild type became more noisy. This phenomenon is commonly observed when tracking the response to selection of a library of mutants[1] and is likely due to the ascension to high frequency of *de novo* beneficial mutations. Here, as selection is intense, the window of time during which the signals we track with the barcodes remain representative of the ones coming from the initial mutations introduced in TEM-1 is fairly short.

We then obtain our estimate for W_i by maximizing the lileklihood above, independently for each mutant i. Due to the presence of binomial coefficients in Equation 11, the likelihood P cannot be computed analytically. To address this issue, we resort to Laplace's method, which assumes that $N_i(0)$ is large, and include Gaussian fluctuations, see *https://en.wikipedia.org/wiki/Laplace%27s_method*). Within this approximation, we write, up to an irrelevant additive constant,

$$\ln P(\{\widehat{N}_i(T_k)\}_k|W_i) \quad = \quad \max_{N_i(0)} \left(\Phi(W_i, N_i(0)) - \frac{1}{2} \ln|\Phi''(W_i, N_i(0))| \right) \, ,$$

where

$$
\begin{aligned}
\Phi(W_i, N_i(0)) \;\; = \;\; & \sum_k \Big[\left(\tfrac{1}{2} + W_i^{T_k} N_i(0)\right) \ln\left(d^k W_i^{T_k} N_i(0)\right) \\
- \;\; & \left(\tfrac{1}{2} + d^k W_i^{T_k} N_i(0) - \widehat{N}_i(T_k)\right) \ln\left(d^k W_i^{T_k} N_i(0) - \widehat{N}_i(T_k)\right) \\
- \;\; & \left(\tfrac{1}{2} + \widehat{N}_i(T_k)\right) \ln\left(\widehat{N}_i(T_k)\right) \\
+ \;\; & \left(d^k W_i^{T_k} N_i(0) - \widehat{N}_i(T_k)\right) \ln(1 - p_k) + \widehat{N}_i(T_k) \ln(p_k) \Big] \, ,
\end{aligned}
\qquad (12)
$$

and Φ'' is the second derivative of Φ with respect to its argument $N_i(0)$. This log-likelihood is then maximized numerically with respect to W_i.

As a check of the procedure, we correctly find that the WT fitness is equal to 2, see Supplementary Figure S10(a). We also checked the validity of our approach in simple cases, for which P could be numerically estimated ($k_f = 1$), see Supplementary Figure S10(b,c). We estimate the uncertainty about the inferred fitness W_i through

$$
\sigma_{W_i} = \frac{1}{\sqrt{\frac{\partial^2 \ln P(W_i, N_i(0))}{\partial W_i^2}}} \, . \qquad (13)
$$

Since $W_{WT} = 2$, and $\frac{\sigma_{W_i}}{W_i} \ll 1$, the standard deviation $\sigma_{\ln(w_i)}$ associated with the relative log-fitness is equal to $\frac{\sigma_{W_i}}{W_i}$.

Definition of the lethality threshold

Mutants were considered lethal under a theoretical lower threshold for log-fitness equal to $-\ln 2$. At this specific value, bacteria do not grow in the solution. In practice, to limit the noise, we used a higher threshold equal to -0.6.

Supplementary Note 2: Computation of MIC.

To compute the MIC the excluded mutants with less than 35 reads at time 0 in replicate 2. Read counts were then turned into bacterial counts by turning them to frequency and multiplying them by the counted number of cells recovered at that concentration. The values were then smooth splined along the log2 of the antibiotic. We added for that the value observed without antibiotic at concentration 0.015625 (as log2(0) is not defined) and added also a value of 0 at a concentration of 64 were no survival was observed.

mod=loess(viability~log2(drug),data=df,span=0.75)

yfit=predict(mod1,newdata=log2(df$drug))

We could then compute the MIC_{mma} with the moment matching approach as mention in the methods section with:

$m = mean(yfit)$

$v = mean(yfit^2) - mm^2$

$MICmma = 12/(1 + vv/mm^2)$

The second measure of MIC is the log 2 concentration of Amoxicillin where only 25% of the mutant population initial survived. For that we did a linear extrapolation between the log2 concentrations where this threshold was passed.

indexfirst=which(yfit<0.25)[1]

$$MIC75 = indexfirst - 1 + (0.25 - yfita[indexfirst - 1])/(yfit[indexfirst] - yfit[indexfirst - 1])$$

Finaly, once these MIC estimates have been computed for all mutants, for each replicat, we performed a principal component analysis with the two metrics:

$Pca1 = prcomp(cbind(MICmma, MIC75), scale = TRUE)$

and took the first component that explained more than 99.9% of the variance as a combined value for log2(MIC).

$MICcombined = Pca1\$x[,1]$

For later analysis the wild type value was subtracted, such that as for fitness, all scores are relative to wild type.

Supplementary Note 3: Computation of epistasis and epistasis type

To compute the epistasis for the different metrics, we used the measure of the double mutant, d, that of single mutants that compose it, s1 and s2 and the wild type value, wt assumed to be 0 later on.

$$epistasis = d + wt - s1 - s2 = d - s1 - s2 \quad (14)$$

When at least one of the values among d, s1, s2 or s1+s2 are below the threshold of functionality, T, the resulting value of epistasis cannot be estimated quantitatively so we set the value of epistasis to 100 to allow easy filtration in the R codes later on. We can however in some cases see the direction it takes. For instance, positive epistasis could be inferred if a double mutant's effect (s1+s2) is predicted to be below T, but is measured double mutants value is above T. We stored that direction of epistasis through a shift around the value 100. In detail:

If both double mutant effect and the sum of single mutant effects are below T, epistasis is set to 100

$if\ (d < T \mid s1 + s2 < T)\ \{epistasis = 100\}$

If the double mutant effect is below T but the sum of the singles is above, there is negative epistasis with an effect of at least -(s1+s2-T). We store it as 100-(s1+s2-T).

$if\ (d < T\ \&\ s1 + s2 > T)\ \{\ epistasis\ = 100 - (s1 + s2 - T)\}$

If the double mutant effect is above T but the sum of the singles is below, there is positive epistasis of at effect at least d-T. We store it as 100+(d-T).

$if\ (d > T\ \&\ s1 + s2 < T)\ \{\ epistasis\ = 100 + (d - T)\}$

To compute the type of epistasis, we first computed the sign by comparing the double mutant effect to the sum of single effects.

$if\ (d > s1 + s2)\ \{sign = 1\}$

$if\ (d < s1 + s2)\{sign = -1\}$

We then computed the number of mutations showing sing epistasis, that is to say changing sign of effect upon addition of the other mutation.

$Reciprocal\_epistasis\_for\_mutation\_1 = test(s1 * (d - s2) < 0)$

$Reciprocal\_epistasis\_for\_mutation\_2 = test(s2 * (d - s1) < 0)$

Where $test(s1 * (d - s2) < 0)$ returns 1 if $s1 * (d - s2) < 0)$ and 0 otherwise.

The value assigned the mutation type is then

$type = sign(1 +\ Reciprocal\_epistasis\_for\_mutation\_1 +$
$Reciprocal\_epistasis\_for\_mutation\_2)$

This means that a value of -3 means reciprocal negative epistasis, -2 negative sign epistasis, -1 magnitude negative sign epistasis, 0 no epistasis, 1 magnitude positive sign epistasis, positive sign epistasis and 3 positive reciprocal sign epistasis. These numbers are used to colour the figures. For fitness and the fractions reported in the main text we also took into account the error in the estimation of fitness to assign to the class 0, mutations for which epistasis was not significant. For that, we computed the standard deviation in epistasis measurement as the square root of the sum of the standard deviation of the double mutant and single mutant fitness estimates

$Epistatis\_sd =\ sqrt(d\_sd\text{^}2 + s1\_sd\text{^}2 + s2\_sd\text{^}2)$

And tested whether the 95% confidence interval of the epistasis measure excluded 0.

Supplementary Note 4: Estimation of the error in Fitness and MIC estimates

To compute the ΔΔG, we need an estimate of the error associated to the MIC or fitness measurement. Using the two replicates, we could observe that the standard deviation of the measure was different between single and double mutants as the former have usually very large counts and therefore more precise estimates. Furthermore, we observed that the standard deviation of the measure was impacted by the mean value of the metric. We therefore computed an estimate of the error for single and double mutants independently, by doing a smooth spline on the measured standard deviation of the metric as a function of the mean value of that metric across the two replicates. We used the function loess in R (span=0.75)

$Sd_{fit} = loess(sd \sim mean, data = single\_mutants \; or \; double\_mutants)$.

$Predicted_{sd} = predict(Sd_{fit})$

Outliers for which the observed standard deviation was more than 7 times larger than the predicted one were excluded from the estimation of ΔΔG.

To be sure that fitness and MIC could be compared, we computed the error of fitness in a similar way. Estimation of ΔΔG using the errors in fitness derive from barcode as explained in Supplementary Note 1 or the one derived from this method that relies on the two replicates gave similar values, so we report in the text the result of the later to have a fair comparison with MIC metrics. The tables fitness or MIC estimates and their standard deviations were then fed a python program to estimate the ΔΔG as presented in the method section.

Supplementary Note 5: Estimation of the error part of the stability model prediction

Using the whole data set, we could estimate an error to the model using a maximum likelihood framework. $\Delta\Delta G$ values were fixed. We estimated that the deviation of the observed log-fitness to the one predicted with the stability model resulted from an overall random deviation from the model. This deviation could be either the same for all pairs of mutations (single-error model) or could be different for residues in contact or not (two-error model). The two-error model was always found to be much better than the single-error one, and produced a higher deviation for residues in contact than for distant residues.

Let $w_{i,j}^{a,b}$ be the experimental relative fitness associated to mutations a, b on sites i, j from the WT, and $\hat{w}_{i,j}^{a,b}$ its model counterpart. Then, for the single-error model:

$$\sigma_m = \sqrt{\frac{1}{N}\sum_{i,j,a,b}\left(\log(w_{i,j}^{a,b}) - \log(\widehat{w}_{i,j}^{a,b})\right)^2}\ , \tag{15}$$

where N is the number of terms in the previous sum.

For the double error model:

$$\sigma_{md} = \sqrt{\frac{1}{N_d}\sum_{i,j,a,b}(1-\delta_{i,j})\left(\ln(w_{i,j}^{a,b}) - \ln(\widehat{w}_{i,j}^{a,b})\right)^2}, \tag{16}$$

$$\sigma_{mn} = \sqrt{\frac{1}{N_n}\sum_{i,j,a,b}\delta_{i,j}\left(\ln(w_{i,j}^{a,b}) - \ln(\widehat{w}_{i,j}^{a,b})\right)^2}, \tag{17}$$

where $\delta_{i,j}$ if the side chains of residues carrying mutation i and j are less than 6Å away and 0 otherwise. In addition, $N_d = \sum_{i,j,a,b}(1-\delta_{i,j})$ and $N_n = \sum_{i,j,a,b}\delta_{i,j}$, $(N = N_d + N_n)$.

We also considered a model with a specific error between two given sites, see Methods:

$$\sigma_{i,j} = \sqrt{\frac{1}{N_{i,j}}\sum_{a,b}\left(\log(w_{i,j}^{a,b}) - \log(\widehat{w}_{i,j}^{a,b})\right)^2}\ , \tag{18}$$

where $N_{i,j}$ is the number of double mutations between the sites i and j.

Supplementary Note 6: Computation of p-value

If we choose as a null hypothesis that there is no relationship between pairs of sites with the largest idiosyncratic epistasis and pairs of sites with the largest Frobenius norm, the probability that among L pairs of sites with the largest idiosyncratic epistasis, c are also present in the L pairs of sites with the largest Frobenius norm follows an hypergeometric distribution with parameters N, L and c, where N is the total number of possible pairs. Therefore, the p-value associated with the observation of c reads

$$p = \sum_{k=c}^{L}\frac{\binom{L}{k}\binom{N-k}{L-k}}{\binom{N}{L}}. \tag{19}$$

Supplementary Note 7: Data and codes

In the zenodo repository (https://doi.org/10.5281/zenodo.18457561) we store the material use to reproduce all data, except the raw data that are store on the ENA PRJEB10446 (https://www.ebi.ac.uk/ena/browser/view/PRJEB10446). The data are proceeded in two steps.

The folder ***1_Preprocessing_Fastq_To_Barcodes_Counts*** stores scripts and data associated with the conversion of the raw reads into mutant frequencies. Associating barcodes to sequences and the counting barcodes frequencies were computed with an R script relying on the use of Mothur: *Processing_BarCodeCoupling_Bioinfo_Mothur.html.* The outputs give the read counts for each bar codes and each time or concentrations (Data092020_FiterA10.csv and CMI_Combo2.csv)

The folder **2_Fitness_MIC_DDG_Epis**, stores data as well as two jupyter notebooks to produce all data from the article. A first notebook 2_Fitness_MIC_DDG.ipynb

Is used to infer fitness, MIC, ΔΔG as well as epistasis. The intermediate files are produced are then used by a second jupyter notebook, 2_Figures_Tables.ipynb, to produce figures, tables, and source data.

Finally, for easier usage by other scientists, out of these files, in the folder **3_User_friendly_data**, we have recomputed some simplified files that provide:

1) For all barcodes the raw counts for fitness and for MIC computation: **BCCountsMIC.csv,**
2) For all genotype the aggregated counts for Fitness and MIC **MutantCountsMIC.csv,**
3) For all genotypes (excluding the one with indels of stop) the fitness and various MIC estimates as well as the predicted impact of mutations with independent and Potts model. Errors are provided for fitness.

   SingleMutantsData.csv, DoubleMutantsData.csv. All column names are explicit. All values are normalized to the wild type value and log for fitness and log2 for MIC. MICmma refers to MIC computed with a moment matching approach, MIC75 refers to MIC computed using the concentration leading to a 75% death rate, MICcombined is derived along the first component of a PCA using the two previous metrics. For the columns categorizing the type of epistasis: 1 refers to positive epistasis, 2 sign positive epistasis, 3 reciprocal sign positive epistasis, -1 negative espistasis, -2 sign negative epistasis, -3 reciprocal sign negative epistasis, and 0 to either no epistasis or undefined epistasis.
4) For all single mutants, the predicted ΔΔG from the two states model is provided for the best fit model whose ΔG_0 is found in supplementary table 16. **DDGSingleMutants.txt**

The multiple sequence alignment for the betalactamase used to train data is Philippon_Data_aligned_Marks_WT.fasta**,** three models are provided: (i) the independent

model (LL_independent_model_single_mut.pkl, LL_independent_model_double_mut.pkl), (ii) the Potts model **(**LL_PLM_model_single_mut.pkl, LL_PLM_model_double_mut.pkl**)**.

1. Couce, A. *et al.* Changing fitness effects of mutations through long-term bacterial evolution. *Science* **383**, eadd1417 (2024).
